# Hormonal Regulation of Oxidative Phosphorylation in the Brain in Health and Disease

**DOI:** 10.3390/cells10112937

**Published:** 2021-10-28

**Authors:** Katarzyna Głombik, Jan Detka, Bogusława Budziszewska

**Affiliations:** 1 Laboratory of Immunoendocrinology, Department of Experimental Neuroendocrinology, Maj Institute of Pharmacology, Polish Academy of Sciences, Smętna 12, 31-343 Kraków, Poland; detka@if-pan.krakow.pl (J.D.); budzisz@if-pan.krakow.pl (B.B.); 2 Department of Biochemical Toxicology, Chair of Toxicology, Medical College, Jagiellonian University, Medyczna 9, 30-688 Kraków, Poland

**Keywords:** mitochondria, oxidative phosphorylation, hormones, brain

## Abstract

The developing and adult brain is a target organ for the vast majority of hormones produced by the body, which are able to cross the blood–brain barrier and bind to their specific receptors on neurons and glial cells. Hormones ensure proper communication between the brain and the body by activating adaptive mechanisms necessary to withstand and react to changes in internal and external conditions by regulating neuronal and synaptic plasticity, neurogenesis and metabolic activity of the brain. The influence of hormones on energy metabolism and mitochondrial function in the brain has gained much attention since mitochondrial dysfunctions are observed in many different pathological conditions of the central nervous system. Moreover, excess or deficiency of hormones is associated with cell damage and loss of function in mitochondria. This review aims to expound on the impact of hormones (GLP-1, insulin, thyroid hormones, glucocorticoids) on metabolic processes in the brain with special emphasis on oxidative phosphorylation dysregulation, which may contribute to the formation of pathological changes. Since the brain concentrations of sex hormones and neurosteroids decrease with age as well as in neurodegenerative diseases, in parallel with the occurrence of mitochondrial dysfunction and the weakening of cognitive functions, their beneficial effects on oxidative phosphorylation and expression of antioxidant enzymes are also discussed.

## 1. Introduction

Maintenance of body homeostasis depends on proper communication between various organs and cells in a living organism, and this communication is in large part achieved by two different yet complementary systems, namely, the nervous system and the endocrine system. While the nervous system is responsible for rapid transmission of information via nerve impulses, allowing quick processing and response to internal and external stimuli, the endocrine system functions by releasing hormones into the bloodstream, which then bind to specific receptors in target cells. Thus, its action is much slower, yet it is able to exert more long-lasting and widespread effects in the body, regulating a plethora of different processes, including development, growth, metabolism, electrolyte balance and reproduction in living organisms [1]. The nervous system and endocrine system are involved in constant reciprocal interactions. On the one hand, endocrine functions are tightly regulated by the central nervous system (CNS), and the hypothalamus is the brain structure that exercises superior control of peripheral hormone secretion from endocrine glands. In response to stimuli from higher brain centers, hypothalamic neuroendocrine neurons release neurohormones (also referred to as releasing hormones) into the hypophysial portal blood to act on cells of the anterior pituitary gland to induce the production of pituitary hormones, which are then transported via the bloodstream to effector endocrine glands such as the thyroid, adrenal glands and gonads or, as in the case of growth hormone or prolactin, act directly on target cells. To maintain the actions of hormones within appropriate boundaries, their release from specific glands is organized mainly in negative feedback loop mechanisms between the hypothalamus/pituitary and selected endocrine glands, which means that hypothalamic and pituitary hormone secretion is generally downregulated by high peripheral hormone levels [2].

On the other hand, both the developing brain and adult brain are target organs for the vast majority of hormones produced by the body, including insulin, glucocorticoids, appropriate sex hormones, and thyroid hormones, which are able to cross the blood–brain barrier (BBB) and bind to their specific receptors on neurons and glial cells. Furthermore, it is now known that some peripheral hormones, such as insulin, insulin-like growth factor-1 (IGF-1) or incretin hormones, are synthetized locally by neurons and glial cells in particular areas of the brain. Hormones ensure proper communication between the brain and the body by activating adaptive plasticity mechanisms necessary to withstand and react to changes in internal and external conditions by regulating neuronal and synaptic plasticity, neurogenesis in the hippocampal dentate gyrus and metabolic activity of brain cells [3]. It is noteworthy that any dysfunction or dysregulation in hormone secretion may severely affect the functioning of the brain and contribute to the progression of different pathologies in the CNS. For example, central insulin and leptin resistance is known to cause cognitive decline [4], whereas chronic and excessive action of glucocorticoids, which under physiological conditions are necessary to generate a proper stress response, may contribute to dendritic shrinkage and debranching and decrease hippocampal neurogenesis, which leads to volumetric changes in this brain structure and may contribute to the development of depressive symptoms [5].

**Figure 1:**
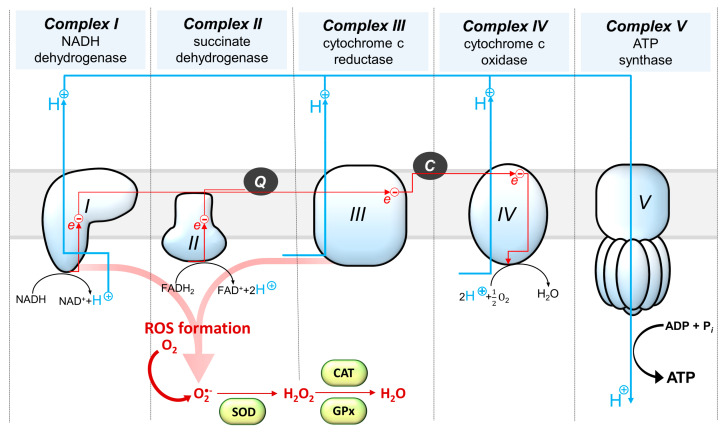
Schematic representation of mitochondrial respiratory chain complexes, outlining the sites of reactive oxygen species (ROS) generation and mitochondrial enzymes involved in the maintenance of proper redox status. Q—coenzyme Q; C—cytochrome C; SOD—Superoxide dismutase; GPx—glutathione peroxidase; CAT—catalase.

Currently, the influence of hormones on energy metabolism and mitochondrial function in the brain has gained much attention, since mitochondrial dysfunctions are observed in many different pathological conditions of the CNS, including neurodegenerative diseases such as Parkinson’s disease and Alzheimer’s disease [6], as well as neuropsychiatric disorders, such as depression [7] and schizophrenia [8]. Mitochondria are organelles that generate most cellular energy in the form of ATP via the process of oxidative phosphorylation (OXPHOS), in which electrons are transferred along the electron transport system (ETS), consisting of four enzyme complexes (I-IV) located in the cristae of the inner mitochondrial membrane. Electron transport across the membrane generates a proton gradient, which is harnessed by complex V of ATP synthase/ATPase to produce ATP from ADP and inorganic phosphate. In addition to their imperative role in energy production, mitochondria are also necessary for regulating calcium homeostasis, programmed cell death and the cell cycle and for the generation and control of reactive oxygen species (ROS) [9]. Complex I and complex III are major sites of ROS generation in the ETS due to electron leakage, and to overcome oxidative stress, mitochondria rely on several defense mechanisms, including antioxidant enzymes such as superoxide dismutase (SOD), glutathione peroxidase (GPx), catalase (CAT) and glutathione, transported from the cytosol to restore redox balance [10] (Figure 1).

To adapt to constantly changing metabolic conditions inside the cell, mitochondria experience a series of dynamic changes to maintain their proper function. First, in response to different cellular stressors, the cell activates the expression of mitochondrial stress response genes, encoding chaperones and proteases, which regulate the folding and degradation of unfolded or misfolded mitochondrial proteins to restore the function of these organelles [11]. Mitochondria also undergo changes in their structure, such as fusion and fission. Fusion is mediated by proteins such as mitofusins 1 and 2 (Mfn1/2) and optic atrophy factor-1 (OPA1), whereas dynamin-related protein 1 (DRP-1) regulates fission of the mitochondria, and it was repeatedly demonstrated that the imbalance between these processes can impair their function, leading to degradation of these organelles [12,13].

The brain is an organ characterized by almost disproportionally high energy demands, and to sustain their proper function, neurons require a constant supply of ATP. Since they do not possess the ability to store glycogen, nerve cells rely mostly on the energetic support of astrocytes, which produce lactate in the process of glycolysis to be transported into neurons, where it is directly utilized in the tricarboxylic acid (TCA) cycle and oxidative phosphorylation to generate energy [14,15]. As postmitotic cells, neurons are also very susceptible to ROS-induced damage, and therefore, any disturbance in OXPHOS efficiency or mitochondrial integrity in the CNS may result in a potentially deleterious effect on brain function. Because direct or indirect regulation of cellular metabolism is an important aspect of many hormone actions, and in recent years it has been proven that some hormones can directly affect brain mitochondria by regulating gene transcription in mitochondrial DNA (mtDNA) [16], a better understanding of the influence of different hormones on the regulation of the OXPHOS process and mitochondrial integrity in the CNS seems to be an important issue, which, however, has not been comprehensively discussed thus far. Therefore, in this review article, we present current findings regarding the influence of signaling mediated by different hormones on mitochondrial respiratory processes in the brain and provide insights regarding various aspects of central hormonal action in the context of the development and treatment of CNS diseases.

## 2. Glucoregulatory Hormones Affect Energy Homeostasis and Mitochondrial Function in the Brain

Whole-body glucose metabolism is regulated by two opposing-acting peptide hormones synthesized by the pancreas, namely, glucagon and insulin. Glucagon is secreted from pancreatic α cells under hypoglycemic conditions to increase blood sugar levels mainly by stimulating the process of gluconeogenesis in the liver and promoting the breakdown of glycogen. On the other hand, insulin, which is secreted from the β cells of pancreatic islets, is the main hormone responsible for decreasing the levels of circulating glucose and participates in the regulation of lipid and protein metabolism. Upon binding to its specific receptor (IR) in target organs, which include liver, skeletal muscle and adipose tissue, insulin activates cellular events aimed at promoting glucose uptake and metabolism, glycogen and lipid synthesis and inhibition of gluconeogenesis. Moreover, peripheral secretion and action of insulin are facilitated by incretin hormones, such as glucagon-like peptide-1 (GLP-1) and glucose-dependent insulinotropic peptide (GIP), which are secreted from enteroendocrine cells in the intestines, in response to the presence of food nutrients [17].

Insulin action is not limited to the periphery. The brain, which was considered for a long time to be an insulin-insensitive organ mainly because of low expression of insulin-dependent glucose transporter-4 (GLUT-4), is now clearly proven to react to physiological concentrations of this hormone. Insulin receptors are widely expressed throughout the CNS and are present at the highest density in brain areas involved in cognitive and autonomic functions and regulation of appetite. Insulin can not only be actively transported across the BBB but can also be synthesized by neurons and, to a lesser extent, by glial cells in various brain regions [18]. In the CNS, insulin action is complementary to its peripheral functions and consists of the maintenance of energy homeostasis via regulation of food intake and feeding behavior by interactions with orexygenic and anorexygenic neuronal populations in hypothalamic nuclei [19]; however, it was also repeatedly demonstrated that insulin is able to support neuronal function in the brain by preventing neuronal apoptosis, promoting synaptic plasticity and affecting neurotransmission by regulating neurotransmitter uptake and receptor density, and therefore, its action is important for proper maintenance of many brain functions, such as mood, cognition, memory, learning and attention [20].

In the CNS, insulin acts alongside insulin-like growth factor-1 (IGF-1), a closely related trophic hormone involved in cell growth, proliferation and differentiation, which in the periphery is produced mostly by the liver and is released into the bloodstream in response to growth hormone (GH) secretion from the anterior pituitary. For example, insulin and IGF-1 can be transported across the BBB and are also locally synthesized in various parts of the CNS [21,22]. IR and IGF-1 receptor (IGF-1R) share high structural and functional similarity and can both be activated by insulin. These receptors are heterodimeric proteins belonging to the tyrosine kinase receptor family, and moreover, they utilize similar downstream signaling routes, namely, the mitogen-activated protein kinase–extracellular signal-regulated kinase (MAPK/ERK) pathway, which regulates cell proliferation and gene expression, and the phosphoinositide 3-kinase/protein kinase B (PI3K/Akt) pathway, which mainly affects metabolic processes and synaptic plasticity in the brain. Despite some differences concerning their role in the development of the organism and slightly different distribution and density between IR and IGF-1R in particular brain regions, the action of both peptides in large part exerts similar biological effects in the CNS [23].

Insulin resistance, defined as impaired or lack of response of target tissues to insulin as a consequence of downregulation of IR or its downstream effector proteins such as insulin receptor substrates (IRS-1 and IRS-2), as well as impaired binding activity of IR, is a relatively common dysfunction, which can lead to the development of metabolic diseases such as type 2 diabetes mellitus (T2DM) and obesity, but impairments in central insulin signaling are also known to reduce synaptic plasticity and cognitive functions and therefore are often linked to the development of neuropsychiatric disorders such as depression [24] as well as facilitating neurodegeneration, which may lead to the progression of dementias, including Alzheimer’s disease [25]. Although the role of brain insulin resistance in the pathogenesis of both depression and Alzheimer’s disease appears to be evident, the exact cellular and molecular mechanisms by which it may affect cognitive, mood and neurodegenerative processes are not sufficiently explained; however, the results of current clinical and preclinical studies emphasize the role of neuroinflammation as well as impaired mitochondrial function and increased oxidative stress as common denominators, linking insulin resistance with the development of both depressive symptoms as well as memory and cognitive deficits in patients [26].

Deficiencies in either insulin or IGF-1 signaling in the brain have been known to cause a decrease in OXPHOS efficiency, and these changes are often clearly correlated with increased levels of oxidative stress markers and altered mitochondrial morphology. For example, the activity of complexes III, IV and V were significantly reduced in streptozotocin (STZ)-treated rats with a simultaneous increase in ROS and NO generation [27]. Transgenic mice with neuron-specific deficiency of the insulin receptor (NIRKO) displayed altered mitochondrial morphology, reduction in the oxygen consumption rate and a decline in protein expression of mitochondrial respiratory complexes in the striatum, also showing signs of oxidative stress-induced damage [23]. Similarly, selective knockdown of IGF-1R in astrocytes not only contributed to impairment of working memory and increased gliosis in mice but also decreased the oxygen consumption rate in primary astrocytic cultures. Astrocytes obtained from mice in IGF-1R knockdown animals were also more susceptible to H_2_O_2_-induced damage and displayed significant impairment in the uptake of glucose and amyloid β (Aβ) [28]. In another study, AAV-induced knockdown of liver IGF-1 gene expression in male mice at the fifth month of age over time resulted in a significant decrease in cortical ATP levels and a reduction in mitochondrial OXPHOS coupling capacity in the hippocampus, which was further associated with an increase in the levels of oxidative stress markers, as well as impairments in hippocampal-dependent spatial acquisition and learning. It is noteworthy, however, that in the same experiments, deficiency in circulating IGF-1 levels did not influence or resulted in opposite effects on mitochondrial respiration in some of the peripheral tissues, since it increased lipid peroxidation in adipocytes and reduced adipose mass, which seem to illustrate well the tissue-dependent pleiotropic action of IGF-1 and differences between its peripheral and central effects [29]. These experiments clearly illustrate that insulin and IGF-1 signaling are necessary for maintaining proper respiratory function and redox status in the brain.

On the other hand, there is also much evidence that insulin and IGF-1 directly influence OXPHOS efficiency and mitochondrial stability in the brain. In numerous in vitro studies conducted on neuronal and glial cell lines and primary cultures, stimulation with insulin or IGF-1 improved mitochondrial respiration, upregulated the expression of mitochondrial complexes and increased ATP production [30, 31, 32]. Similar results were also observed in in vivo experiments, and most of the current data concerning the direct central action of insulin energy metabolism and mitochondrial function come from experiments with intranasal administration of insulin, which enables us to study its direct action in the brain and omit peripheral effects of this hormone. Although the precise mechanisms by which insulin enters the CNS via this administration route have not yet been entirely explained, it has been demonstrated that its intranasal application can prevent neuroinflammation and nerve damage and improve memory and cognition in preclinical and clinical studies [33, 34, 35]; furthermore, it was able to reduce weight loss in humans in a sex-dependent manner [36]. Intranasal insulin was shown to ameliorate ATP synthesis, improve calcium homeostasis, reduce ROS formation and modulate mitochondrial biogenesis by upregulating the expression of peroxisome proliferator-activated receptor gamma coactivator 1-α (PGC1α) in experimental mouse models of diabetes and Parkinson’s disease [31, 37]. The exact mechanism by which insulin administration is able to stimulate energy generation in the brain is still not clearly understood; however, recent reports indicate that it may be associated with modulating the activity of ion channels in mitochondrial membranes. Improvement of complex I and IV activity, redox status and membrane potential after intranasal insulin administration in STZ-induced early type 2 diabetic mice was associated with an increase in the activity of the mitochondrial ATP-sensitive large conductance Ca^2+^-activated potassium channel (mitoBK_Ca_) along with upregulation of the expression of its β2 subunit [38]. It was also recently demonstrated that insulin can improve the mitochondrial stress response, since short-term intranasal insulin treatment increased the expression of MSR-related genes, including *Atf4*, *Chop*, *Hsp60*, *Hsp10*, *ClpP*, and *Lonp1,* in the hypothalamus of high-fat diet (HFD)-fed C57BL/6N mice, which was associated with reduced food intake and body weight development in experimental animals [30]. Experimental data concerning the direct influence of central insulin action on the brain in energy metabolism are not limited to rodents. In a clinical study performed on a group of healthy young volunteers in which cerebral energy metabolism was assessed with ^31^P magnetic resonance spectroscopy, intranasal insulin application contributed to the increase in the levels of ATP and phosphocreatine in the brain [39].

Peripheral administration of many T2DM drugs, which act as insulin-sensitizing agents, was often shown to not only reverse central insulin resistance but also to alleviate the associated brain mitochondrial dysfunction and improve respiratory function and redox capacity in the CNS. Metformin for example is known to act as an inhibitor of the mitochondrial complex I, which is a notable site for ROS production (Figure 1) and this feature is regarded as an important mechanism of the antidiabetic action of this drug [40]. Although its intracerebroventricular administration at high doses was previously shown to cause profound neurotoxic effects in the brain and increase in oxidative stress [41], metformin is generally known to exert a neuroprotective effect and has many times been shown to have neuroprotective and antioxidant properties in the brain. For example, the inhibition of complex I activity by metformin and phenformin was associated with a reduction in the opening of mitochondrial permeability transition pore (mtPTP), which prevented ischemia-induced damage in cultured rat brain slices [42]. In HFD-induced animal models of insulin resistance, metformin treatment resulted in alleviation of oxidative stress [43] and recently it was also shown to increase ATP production in the brain [31]. In HFD-fed rats, an improvement in brain mitochondrial viability, function and redox capacity was observed also after chronic treatment with other antidiabetic drugs, including peroxisome proliferator-activated receptor-γ (PPAR-γ) agonists [44] and inhibitor of sodium glucose cotransporter-2 (SGLT-2) inhibitors [45].

Among insulinotropic compounds used in the treatment of T2DM, the administration of which is known to have a beneficial effect on the brain energy expenditure, incretin-based drugs deserve special attention, namely because they act by enhancing or mimicking the action of endogenous incretin hormone glucagon-like peptide-1 (GLP-1). GLP-1 is synthetized by the intestinal L-type cells but also in the brain, mostly by neurons in the nucleus of solitary tract (NTS) in the caudal brainstem. In the body, GLP-1 is an important regulator of energy homeostasis, secreted from the gut in response to the presence of food nutrients. Its role includes stimulating insulin secretion and potentiation of peripheral insulin action by binding with GLP-1 receptors (GLP-1R) located in many tissues in the periphery, as well as the CNS [46]. GLP-1 in the brain acts as an anorexygenic peptide, which decreases food and water intake and food-related reward, and although it can be actively transported by BBB, it is characterized by a very short half-life and thus peripherally synthetized peptide can act only locally [47,48] and has a rather low ability to activate GLP-1R in distant brain areas [49]. Instead, preproglucagon (PPG) expressing neurons of the NTS are the prime source of central GLP-1 and project to various brain areas involved in the regulation of feeding behavior, including hypothalamus, midbrain, amygdala, hippocampus and prefrontal cortex [50,51]. It was for a long time postulated that the release of GLP-1 from NTS neurons is triggered by its peripheral secretion via vagal afferent signals resulting from stimulation of GLP-1R located in the vagus nerve, however recent report shown that PPG expressing neurons possess a very limited neuroanatomical connection with GLP-1R expressing vagal neurons, instead receiving input mostly from neurons, which express oxytocin receptor (OXTR), thus suggesting that central and peripheral GLP-1 signaling may facilitate its anorexygenic effects via two independent circuits [52]. Although the main central role of GLP-1 consists of the regulation of feeding behavior, studies conducted over the years elucidated more complex functions of GLP-1 in the brain, including the involvement in regulation of endocrine function including stress response, by stimulating hypothalamic neuroendocrine neurons in the PVN [53].

GLP-1 receptors are widely expressed throughout the CNS and are present both on cell bodies and fiber terminals of neurons as well as in astrocytes and microglia [54,55,56]. Incretin-based therapeutics, which employ either stable agonists of GLP-1 receptor (GLP-1R) or inhibitors of dipeptidyl peptidase-4 (DPP-4), which is the enzyme responsible for cleavage and inactivation of circulating GLP-1 in the body demonstrated neuroprotective and neurotrophic properties, including improvement of synaptic plasticity and reduction of neuroinflammatory processes. Treatment with therapeutic GLP-1R agonists, such as exenatide and liraglutide, resulted in increased neurogenesis in the dentate gyrus [57], and some data suggest that GLP-1R can exert protective effects on brain mitochondria, which were mediated by the activation of the PI3K/Akt pathway and increased levels of the antiapoptotic protein Bcl-2 [58]. GLP-1R agonists are therefore often considered for the treatment of neurodegenerative diseases as well as depression [58,59], especially considering the fact that these peptide drugs display significant efficacy in crossing the blood–brain barrier [60,61].

To date, there are few data describing the effects of GLP-1 signaling on energy metabolism and mitochondrial function in the brain. For example, in HFD-induced model of insulin resistance in rats, treatment with DPP-4 inhibitors vildagliptin or sitagliptin improved cognitive function and simultaneously reduced ROS production and mitochondrial swelling and decreased the membrane potential [62]. Treatment with GLP-1R agonist liraglutide was shown to improve motor functional recovery and increased axonal sprouting of the cortical neurons in a mouse model of focal cerebral ischemia, which was associated with the enhancement of ATP generation, increased activity of mitochondrial complex I and three key enzymes of the TCA cycle, i.e., isocitrate dehydrogenase, α-ketoglutarate dehydrogenase, and succinate dehydrogenase [63]. GLP-1 signaling may also influence mitochondrial dynamics and integrity in both glial cells and neurons, since astrocyte-specific knockout of GLP-1R in mice was shown to increase mitochondrial fragmentation in the hypothalamus [56]. It was also demonstrated that treatment with liraglutide was able to mitigate mitochondrial fragmentation by increasing the levels of Mfn-2 and OPA-1 and at the same time intensify the phosphorylation of DRP-1, thus promoting mitochondrial fusion over fission, which prevented hippocampal neuronal loss in a transgenic 5 × FAD mouse model of Alzheimer’s disease (expressing human APP and PSEN1 transgenes) [64]. In another study involving a 5 × FAD mouse model, liraglutide promoted aerobic glycolysis in astrocytes, thereby improving their ability to support neurons with energy substrates, which resulted in their increased survival and axonal growth. This mechanism involves the activation of the PI3K/Akt pathway, and astrocytic OXPHOS efficiency is significantly reduced and contributes to decreased generation of ROS [65]. Stimulation of GLP-1R may also prevent oxidative stress in microglia. As shown in an in vitro study conducted on a BV-2 microglial cell line, activation of GLP-1R and glucose-dependent insulinotropic polypeptide receptor (GIPR) reduced apoptotic cell death, increased the synthesis of neurotrophic factors and alleviated oxidative stress by inhibiting the accumulation of ROS and the release of nitric oxide (NO), as well as by upregulating the expression of antioxidative enzymes such as GPx1 and SOD1 [55].

## 3. Role of Thyroid Hormones in Brain Metabolism Regulation: Focus on Changes in Oxidative Phosphorylation

The synthesis and secretion of thyroid hormones (THs) are regulated by a negative feedback mechanism that involves thyroid-stimulating hormone (TSH), produced by the anterior pituitary gland, and thyrotropin-releasing hormone (TRH), secreted by the hypothalamus. TSH acts on the thyroid gland to induce the production of the two main THs, thyroxine (T_4_) and triiodothyronine (T_3_), whose endocrine activity is responsible for the regulation of cell energy metabolism and growth and responds to stress because they act on almost all nucleated cells [66,67]. THs are extremely important in the early developmental processes and maturation of the brain. They do not regulate early neuronal processes such as neuronal induction but affect its architecture and functions, including neuronal processing and integration, neuronal and glial cell proliferation and differentiation, myelination, and synthesis of enzymes essential for neurotransmitter formation.

T_4_ and T_3_ are transported to the brain by specific transmembrane transporters, namely, monocarboxylate transporter 8 (Mct8) and the organic anion transporter polypeptide 1c1 (Oatp1c1). The biologically active thyroid hormone T_3_ in the brain derives only 20% from the periphery and is mainly produced locally in the brain by 5′-deiodination of T_4_, mediated by deiodinase 2 (Dio2) in astrocytes, in proportions that depend on the developmental stage. The actions of THs are the result of their interactions with nuclear receptors—thyroid hormone receptor (TR) α and β. The TR subtypes are expressed in the brain from early development and either repress or activate gene expression [68]. An altered supply of thyroid hormones in the developing brain, caused by reduced production of THs by the thyroid gland or insufficient THs uptake by the target cells, causes hypothyroidism, which can lead to mental retardation and neurological deficits [69,70]. It also turns out that the proper levels of these hormones play an important role in the maintenance of adult brain function, and disturbances in TR actions in the adult brain may result in consistent behavioral changes, mood and behavior and lead to psychiatric manifestations [71]. The involvement of T_3_ in the regulation of development, growth and metabolism suggests that it controls a broad spectrum of pathways, one of which is mitochondrial activity [72].

The search for new molecular mechanisms that precisely describe THs action is important because thyroid diseases are currently one of the most common chronic disorders and are relatively easily treatable, but if undiagnosed or left untreated, they can have harmful adverse effects, including long-term psychological consequences. Hypothyroidism and hyperthyroidism are most often effects of pathological processes within the thyroid gland in iodine-replete populations and have mainly autoimmune bases. Hyperthyroidism refers to the condition of increased thyroid hormone synthesis and secretion from the thyroid gland, whereas hypothyroidism is a pathological state with thyroid hormone deficiency [73,74]. Both of these conditions increase the risk for important health issues concerning, among others, CNS dysfunction [75].

Despite frequent cases of hypothyroidism in the clinic, little is known about the impact of hypothyroidism on the factors regulating mitochondrial OXPHOS efficiency in the adult brain, probably because for a long-term adult brain was thought to be resistant to thyroid hormones. Proper levels of THs maintain mitochondrial integrity, and it is known that hypothyroidism alters mitochondrial morphology, leading to their enlargement, increased vacuolization, a decrease in the number of cristae and the loss of transmembrane potential in the cerebellum of neonates [76]. Previous studies carried out in the 1950s demonstrated that treatment of rats with thyroid hormone during the prenatal and early postnatal periods increased oxygen consumption [77,78], which indicated that CNS mitochondria are sensitive to T_3_ at the early stage of development, in agreement with the observed neurological symptoms, such as marked retardation of hypothyroidism during development.

Recent studies concerning the early development period also confirmed these observations. Thyroidectomized fetal sheep were characterized by diminished complex I-linked respiration and complex I abundance and, as a compensatory mechanism, enhanced PGC1α expression simultaneously with thyroid hormone receptor β upregulation in the cortex, while in the cerebellum, hypothyroidism reduced complex I, II and complex II-linked respiration with no impact on ETS complexes. The observed changes in mitochondrial processes were correlated with weakened myelination, which may cause neurodegenerative changes [79]. Additionally, a study on rodents showed a decrease in the OXPHOS rate (when NADH-generating substrates were added) and complex I and III activity in the cerebral cortex and striatum of hypothyroid Wistar rat neonates but not in the hippocampus, cerebellum, thalamus, mid brain or brain stem [80]. The levels of mitochondrially encoded transcripts were diminished only in the brain structures where mitochondrial activity was decreased; therefore, these structures have been recognized as “sensitive” to thyroid hormones, but the lack of effect of thyroid hormones on structures such as the hippocampus, which have functional thyroid hormone receptors, may arise from the fact that the regulation of transcription by T_3_ receptors requires various coregulators, levels of which may vary in different tissues [81]. The impact of hypothyroidism on brain metabolism in these “insensitive” structures in adult animals seems to be similar. In the hippocampus of Wistar rats treated with propylthiouracil (PTU), there were no changes in the levels of proteins forming respiratory chain complexes, and changes in mitochondrial respiration in isolated mitochondria from hippocampi were minor [82]. However, in the same animals, hypothyroidism induced by propylthiouracil resulted in reduced levels of proteins such as complex II and ATP synthase in the frontal cortex, and it was connected with functional changes in mitochondria: intensification of proton leakage across the mitochondrial inner membrane and a reduction in the capacity of the mitochondrial ETS [82].

Currently, research increasingly differentiates the function of mitochondria depending on their location in nervous tissue and distinguishes two major populations of mitochondria: those derived from neuronal and glial cell bodies (CM) and synaptic mitochondria (SM) versus those derived from nerve terminals. Zhuravliova et al. [83] demonstrated elevated redox level of copper of cytochrome oxidase and increased production of peroxides in the presence of substrates of complex I (glutamate and malate) in the CM of hippocampi of hypothyroid (methimazole-treated) rats, whereas succinate-induced H_2_O_2_ release was diminished. Furthermore, previous data [84,85] showed that in the CM of hypothyroid neonates, there was diminished activity of complex I and reduced transmembrane potential, rate of oxidative phosphorylation and oxygen consumption. Moreover, the observed effects were correlated with decreased free mitochondrial biogenesis in the CM of the cerebral cortex, and it is hypothesized that the impact of THs on free mitochondrial biogenesis could underlie their effects on respiration [85]. Interestingly, mitochondrial respiration changes in the abovementioned studies [83,84,85] were observed in CM but not in the synaptosomal mitochondrial fraction. Moreover, in CM mitochondria, the aerobic glycolysis process (measured as formation of lactate) was increased, which was connected with hexokinase—initial, rate-limiting enzyme of glycolysis located on the mitochondria outer membrane—activity enhancement. Adaptive activation in glycolytic metabolism was not observed in SM, which shows that synaptic mitochondria differ in their sensitivity to energetic dysregulation in hypothyroidism [83]. Much less research concerns metabolic changes in the brain through the course of hyperthyroidism. These are mainly studies from the second half of the 20th century showing that Wistar rats injected with a thyrotoxic dose of T_3_ exhibited slight effects in the brain when compared to the liver or kidney. Impaired oxidation of succinate seems to be a common mechanism of T_4_ in all examined tissues [86].

Brain mitochondria are targets of THs and were confirmed by experiments involving T_3_ and T_4_ administration. T_3_ can counteract unfavorable changes observed in hypothyroidic mitochondria in the brain by normalizing diminished respiratory processes and regulating mitochondrial transcript (ND4, 12S rRNA, 16S rRNA and Cox III) levels. High, supraphysiological doses of T_3_ that were used caused all measured factors to return to the control state 72 h after T_3_ treatment [87]. In peripheral tissues (i.e., in the liver), much lower doses of T_3_ enhanced oxygen consumption well above the control [88]. Studies on adult thyroidectomized rats proved that brain mitochondrial respiratory activity could be stimulated by T_3_ and T_4_ but with different effects depending on the substrate used [89]. Adding glutamate with T_3_ and T_4_ caused hyperstimulation of respiratory activity, whereas pyruvate + malate normalized these process rates only at the high dose. Succinate together with TH stimulated respiration but to the control level, while in the case of TH treatment together with ascorbate + N,N,N′,N′-tetramethyl-p-phenylenediamine (TMPD), even the lowest dose of T_3_ stimulated respiration, whereas T_4_ normalized the hypothyroidism-induced changes only at high doses [89]. This study suggested that the effects of THs on respiratory processes in the brain are substrate-specific and dose-dependent. Additionally, both examined THs increased the level of cytochrome oxidase (cytochrome *aa3*). Other authors also demonstrated that precursor polypeptides of the cytochrome *aa3* complex accumulate even under hypothyroidic conditions and that THs regulate synthesis and subunit assembly to create a functional complex [72, 89, 90].

THs augmentation is also one of the recommended treatment strategies for nonresponsive bipolar mood disorders. In clinical studies, L-T_3_ was preferred over L-T_4_ for the combined therapy of acute depression and the prevention of recurrent episodes in bipolar disorder with rapid phase change [91]. Despite the irrefutable evidence that additional therapy with THs is effective in the treatment of depression, for many years, the main mechanism that has been proposed was only the action of THs on the intensification of central serotoninergic neurotransmission and the regulatory function of the β-adrenergic receptor [75]. Recent findings have shown that it is possible that the mechanism of the antidepressant action of these hormones can be different. L-T_4_ improved depressive symptoms, which were strongly correlated with metabolic changes in the brain structures [91]. Studies using positron emission tomography (PET) with [F-18] fluorodeoxyglucose demonstrated that even in euthyroid patients with bipolar disorder, adjunctive treatment with L-T_4_ led to a reduction in the relative activity of limbic and subcortical structures [92]. Animal research on L-T_4_ and/or venlafaxine treatment has also shown specific effects on glycolysis and oxidative phosphorylation in the brain [93]. Venlafaxine is a selective norepinephrine and serotonin reuptake inhibitor often used in the clinic, and in the mentioned study, it was administered together with L-T_4_ to hypothyroidic Wistar-Kyoto rats, which are considered to be a model of depression [94]. The data demonstrated that the most beneficial effect of the combined treatment was glycolysis and the transition from glycolysis to the Krebs cycle because both drugs normalized unfavorable changes in pyruvate dehydrogenase and pyruvate expression caused by depression and hypothyroidic conditions in the frontal cortex. In this study, neither the antidepressant drug nor L-T_4_ improved the changes in the expression of mitochondrial respiratory chain complexes [93]. Therefore, it seems that perhaps the mitochondrial complexes in the brain in coexisting depression and hypothyroidism are not sensitive to thyroid-adjunctive therapy.

## 4. Insights into Glucocorticoid Effects on Brain Oxidative Phosphorylation Processes

Mitochondria mediate the stress response partially by sensing the levels of glucocorticoids [95]. The stress response is characterized by the release of stress mediators-the classic hormones of the stress system (corticotropin releasing hormone (CRH), adrenocorticotropin (ACTH), glucocorticoids (GCs), several neurotransmitters (catecholamines: adrenaline and noradrenaline), cytokines and growth factors. A large amount of energy is required for this system to function properly; therefore, mitochondria are primarily responsible for supplying immediate fuel and support for the ‘fight and flight response’ in the cells and tissues by oxidizing the substrates that are made available by stress hormone-induced mobilization [95]. After the occurrence of a stress stimulus, the neuropeptide CRH, synthetized in the hypothalamic paraventricular nucleus (PVN), is released into portal vessels and transported to the anterior pituitary, where it stimulates the synthesis and secretion of ACTH [96,97,98], which reaches its target organ, the adrenal cortex, via the blood circulation and stimulates the synthesis and secretion of GCs [99].

GCs, which are synthesized in the adrenal cortex from cholesterol, play a major role in survival during stress. GCs can cross plasma membranes by passive diffusion and act through two related steroid hormone receptors, mineralocorticoid receptors (MRs) and glucocorticoids receptors (GRs). After binding with ligands, the receptors undergo conformational rearrangements, dissociation from the chaperones, homodimerization and translocation to the nucleus. In the nucleus, both MRs and GRs act as homodimeric transcription factors that bind to glucocorticoid response elements (GREs) in the promoter regions of GC-sensitive genes, and GRs, as monomers, can also directly regulate the action of other transcription factors [100,101,102].

**Figure 2:**
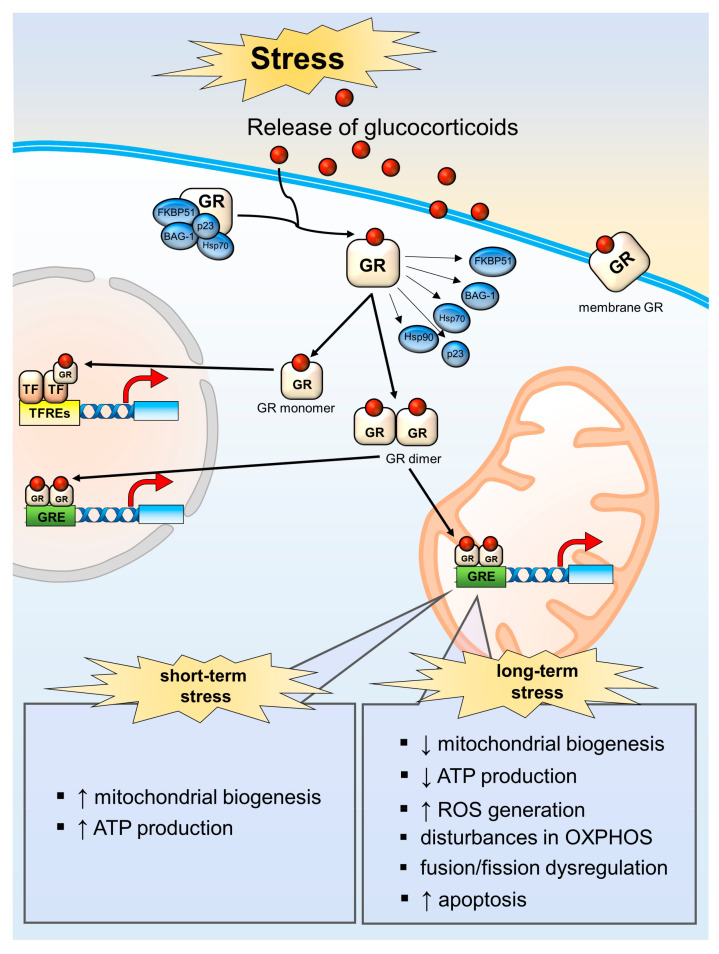
A schematic diagram illustrating the effects of short-term and long-term effects of glucocorticoids on mitochondrial function. GR—glucocorticoid receptor; GRE—glucocorticoid responsive element; TF—transcription factors; TREs—transcription factor responsive elements; FKBP51, BAG-1, Hsp-90, Hsp-70, p23—glucocorticoid receptor co-chaperones: FKBP51—FK506 binding protein 5; BAG-1; Co-chaperone 1Hsp-90—heat shock protein 90; Hsp70—heat shock protein 70; p23—co-chaperone protein p23.

In addition to this well-described molecular mechanism of GCs action and their regulatory function in the stress system by maintaining molecular, cellular and systemic homeostasis in nerve cells [103], GCs are increasingly gaining interest as factors that directly or indirectly influence mitochondrial function. The regulation of mitochondrial function by a stress stimulus depends on its duration and intensity: GC release associated with a short-term stimulus leads to the most beneficial effects: mitochondrial biogenesis enhancement and enzymatic activity of OXPHOS intensification. Shortly after treatment with different GCs (corticosterone, dexamethasone, cortisol, or prednisolone), there is a decrease in oxygen consumption [104,105]. This effect is probably maladaptive and connected with direct regulation of mitochondrial genes [106] but may also have nongenomic action. Chronic exposure to GCs has a strong effect and can cause accumulation of GR in mitochondria, respiratory chain dysregulation, disturbances in fusion–fission processes, ROS generation, and apoptotic processes. However, what processes take place depends on the target tissue and its energy demand under stress conditions [95,107]. It seems that decreased oxygen consumption after a short stress stimulus, which turns excessive with prolonged stress, is an adaptive mechanism connected with the enhancement of glycolysis at the beginning and then the necessity of paying off metabolic debt [108] (Figure 2).

The GR is present in the mitochondria of many cells [109,110]. In addition to its action in the nucleus, it also induces changes in the mitochondrial genome, which was proven by studies on GR import/export in mitochondria [111]. GR binds to six potential mitochondrial GREs: four localized within the COX1 and COX3 genes and two within the D-loop region. COX1 and COX3 encode the catalytic subunits of cytochrome c, the terminal oxidase of the mitochondrial electron transport chain [112]. Transfection of mitochondrial GRE constructs into LATK cells showed that they were dexamethasone-responsive, which supports the hypothesis that GCs exert their action on mitochondrial gene transcription [113]. The effects of a glucocorticoid receptor agonist, dexamethasone, on mitochondrial changes in the brain seem to be most interesting and relevant to metabolic studies on the brain. Dexamethasone, a commonly used synthetic glucocorticoid in the clinic, is characterized by potent anti-inflammatory properties and higher glucocorticoid activity than the natural human glucocorticoid cortisol. It is widely used in the treatment of several disorders, i.e., skin and pulmonary diseases, low birth weight in infants, rheumatoid arthritis [114,115,116] and when there is a risk of a premature delivery recommend prenatal steroid therapy in every case of threatened premature birth between 24 and 34 weeks of pregnancy. In such cases, synthetic glucocorticoids (sGCs) are administered to accelerate the maturation of alveolar epithelium and to stimulate respiratory system maturation in the fetus [117]. Although dexamethasone is commonly used to limit the effects of underdevelopment of the lungs in premature infants, it is known that this treatment option leads to an increased incidence of short-term neurodevelopmental delay. Unfortunately, little is known about the impact of dexamethasone on long-term neurodevelopmental processes in the brain, but the risk of serious adverse neurodevelopmental effects remains after prenatal as well as postnatal corticosteroid treatment [118]. The active form of dexamethasone is able to cross the placenta, explaining why it is a widely used glucocorticoid as a factor triggering the maturation of lung and other tissues in the fetus [117]. It is known that mitochondrial maturation, among other factors, is controlled by steroid hormones, i.e., they are involved in regulatory processes of perinatal mitochondrial maturation in the kidney [119]. In the rat brain, oxidative metabolism maturation occurs during the late gestation period and is correlated with an increase in plasma corticosterone levels [120]; thus, prenatal steroid therapy may induce changes in mitochondrial respiratory activities in the fetal brain. Four doses of dexamethasone administered to pregnant dams at an interval of 12 h (until 12 h before each measurement) led to precocious maturation of mitochondrial respiratory function, manifested by an increase in ADP-stimulated respiration, uncoupled respiration, and respiratory control ratio (RCR) in the fetal forebrain at Day 16 of gestation [121]. These processes may be essential for proper brain function of the newborn because mitochondrial respiratory process enhancement is required immediately after birth to maintain high-energy phosphate concentrations [122]. Studies on the effect of repeated exposure to dexamethasone on the brain mitochondrial oxidative respiratory chain (with the use of different substrates: glutamate, pyruvate + malate, succinate and ascorbate + TMPD) in developing and adult rats at different postnatal stages revealed that administration of this synthetic glucocorticoid stimulated state 3 respiration rates in growing rats (2, 4 and 5 weeks old) in an age-dependent and substrate-specific manner, whereas in adults, state 3 was significantly inhibited [123]. Moreover, injections with dexamethasone led to significant mitochondrial uncoupling with all substrates tested in 3-week rats [123]. This critical period, when the rats switch from mother’s milk to a solid diet, is stress nonresponsive because the plasma corticosterone level is low [124], and it is possible that exposure to excessive dexamethasone levels can lead to its accumulation, thereby resulting in uncoupling of mitochondria. Surprisingly, the levels of cytochrome *aa3 and b* and ATPase activity decreased significantly after dexamethasone in all animals: young and adult [123]. This may be the result of dexamethasone action as an enhancer of the intrinsic rate of electron transfer and its negative impact on peptides encoded by mtDNA [125].

Another comparative study investigated the in vitro effects of four different glucocorticoids, dexamethasone, prednisolone, triamcinolone and hydrocortisone, on mitochondria isolated from the rat brain. Measurements showed that the observed decrease in the respiration control ratio was induced by all glucocorticoids, which was the effect of significant inhibition of state 3 and complex V activity and changed proton fluxes through the mitochondrial inner membrane [104]. This study showed that common action of glucocorticoids on brain metabolism is the decrease in oxygen bioavailability at the inner membrane and inhibition of complex V. In this research, the authors indicate that the unique action of acute dexamethasone treatment, in comparison to other glucocorticoids, was the inhibition of complex I activity and partial reversion of the inhibitory action of the antagonist of complex I–rotenone, resulting in inhibition of ROS generation (superoxide anion radical production) [104]. However, the study was conducted in vitro and with the use of acute administration; thus, conclusions about the therapeutic effects of glucocorticoids cannot be drawn.

Glucocorticoids and the stress hormone system have also been highly implicated in the pathophysiology of many diseases, i.e., depression. Impaired GR function and feedback mechanisms and elevated raised cortisol/corticosterone levels are widely observed in the course of this disease [126,127]. Animal models of repeated stress/glucocorticoid administration have provided much data about mechanisms that are connected with altered glucocorticoid action, i.e., changes in learning-induced plasticity [128], but since it is known that mitochondrial trafficking is arrested by glucocorticoids and these alterations lead to energy deficiency, particularly for synaptic transmission, this pathway is gaining attention [129]. Repeated corticosterone (CORT) injections mimic the behavioral and neurochemical manifestations of depression in rodents. CORT effects are manifested by the increased immobility time measured in the forced-swim test [130,131], suppression of neurogenesis processes in the hippocampus [132], and a volume reduction in this brain area [133], but the mechanism by which the underlying CORT regulates neuron functions has not been fully elucidated. Experiments on mouse neural stem cells demonstrated that CORT administration to cultures significantly changed the proteomic profiles of neurons, which has an impact on intracellular pathways of transmission, among which the mitochondrial oxidative respiratory chain was extensively examined. It was shown that CORT inhibited oxidative phosphorylation in mitochondria by affecting mainly NADH-related enzymes, NADH dehydrogenase (ubiquinone) (Nduf), ubiquinol-cytochrome c reductase complex (Uqcc) and mitochondrial ribosomal protein (Mrp), which disrupted energy metabolism in neurons in vitro and simultaneously disturbed their function [134]. In vivo studies also showed a reduction in oxidative phosphorylation in the brain but at different stages of metabolism, in site-specific uncoupling and in an age-dependent manner [105].

Mitochondria, as highly dynamic organelles, respond to glucocorticoids, which influence their morphology, localization, and removal/replacement and can impair mitochondrial homeostasis and quality control [129]. Usually, various stress conditions intensify mitochondrial fusion in tissues and cells. Energy demand in neurons also results in fragmentation of mitochondria into shorter globular forms. Fission processes are necessary for the removal of damaged mitochondria from the cells, and it was also demonstrated that they are elevated under glucocorticoid action. However, the glucocorticoid-enhancing effect on mitochondrial fission is still controversial [129]. An in vitro study using the SH-SY5Y neuroblastoma cell line showed that dexamethasone may cause mitochondrial dysfunction by increasing the levels of the fission proteins DRP1 and FIS1, enhancing fusion processes by increasing Mfn1 and OPA1 expression, thus causing disturbances in mitochondrial dynamic processes, oxidative stress production, impairment of the generation of energy needed for metabolism and decreased ATP content [135]. Furthermore, this study revealed that an imbalance in mitochondrial dynamic processes caused by dexamethasone is involved in the inhibition of neuronal proliferation [135]. The local energy demand may also cause controlled mitochondrial trafficking, but in neurons, many of them remain stationary because of the high need for ATP. Long-lasting exposure to GCs promotes inhibition of mitochondrial motility, which induces a depression-like phenotype in mice, providing further evidence that energy deficiencies in the brain cause disturbances in neurotransmission and behavior [129].

## 5. Sex Hormone Regulation of Oxidative Phosphorylation in the Brain

Steroid hormones, including estrogens, progesterone and androgens, which cross the blood–brain barrier and/or are synthesized in the CNS, are significantly involved in the regulation of the functions and survival of nerve cells [136]. Many of these compounds have been shown to have neuroprotective properties, and therefore lowering their levels in the brain can play an important role in brain aging and the pathogenesis of some neurodegenerative and neurological diseases [137]. In addition to the classic steroid hormones, neurosteroids, which are synthesized in the brain, both by neurons and glial cells, regulate many functions of CNS cells [136]. Neurosteroids act mainly by activating membrane-associated signaling pathways in the CNS, but some of them may also bind to nuclear receptors and regulate gene expression [138]. Classic neurosteroids that are precursors or metabolites of steroid hormones, such as pregnenolone, dehydroepiandrosterone and allopregnanolone, modulate neurotransmission mainly by acting as allosteric modulators of NMDA or GABA_A_ receptors [139]. Currently, an increasing amount of data points out that mitochondrial function is a potential target for both sex steroid and neurosteroid action [140]. Maintaining the correct energy balance of mitochondria in the CNS is particularly important because nerve cells, especially neurons, need large amounts of ATP to sustain the plasma membrane ionic gradients for proper neural excitability and the process of neurotransmission.

### 5.1. The Influence of Ovarian Hormones on Oxidative Phosphorylation

The beneficial, neuroprotective effects of the female sex hormones 17ß-estradiol (E_2_) and progesterone are relatively well understood, and currently, substantial amounts of data show that the action of these hormones on mitochondrial function is important in this effect. Estrogen action is mediated via genomic (activation of estrogen receptor ERα and ERβ) and nongenomic mechanisms (via membrane-bound ERα and ERβ and G protein-coupled estrogen receptor). Many studies, conducted mainly in rodents, have shown that administration of estrogen, especially 17ß-estradiol, modulates cholinergic and dopaminergic transmission, increases neurotrophin synthesis, enhances cognition and exerts neuroprotective effects. Studies on the effect of E_2_ on metabolism in the brain have shown that this hormone intensifies almost all stages of energy transformation [141]. E_2_ administration prevents ovariectomy-induced reduction in the levels of glucose transporters (GLUT-1, GLUT-2, GLUT-4), increases activity of glycolytic enzymes (hexokinase, phosphofructokinase, pyruvate kinase), the enzyme linking glycolysis to the TCA cycle (pyruvate dehydrogenase), TCA enzyme (aconitase) and enhances oxidative phosphorylation. In the estrogen regulation of mitochondrial energy production, intensifying hexokinase activity may be an important mechanism leading to increased production of ATP [141]. Hexokinase can bind with voltage-dependent anion channels (VDACs) on the mitochondrial outer membrane and thus bind glycolysis with oxidative phosphorylation. Moreover, since E_2_ activates not only hexokinase but also Akt kinase, which regulates the binding of hexokinase with VDAC, the connection of glycolysis with ATP synthesis can be enhanced by estrogen in various ways. It was also shown that administration of E_2_ increases the expression of complex I, complex IV and complex V (ATP synthase). In line with these changes, E_2_ increases the maximal mitochondrial respiratory rate in neurons and glial cells and induces an increase in ATP production [141]. The enhancement of oxidative phosphorylation by 17ß-estradiol is due to its action through both ERα and ERβ receptors; however, ERβ stimulation produces a stronger effect. Intensifying the oxidative phosphorylation process by E_2_ and simultaneous stimulation by this hormone of the expression of some antioxidant enzymes (inter alia peroxiredoxin 5 and SOD) appear to be the main mechanisms underlying its neuroprotective effects, including a protective effect on β-amyloid-induced injuries.

Epidemiologic studies show that the lack of ovarian hormones in women during menopause exacerbates age-related brain changes and increases the risk of neurodegenerative disease development, and these alterations correlate with reduced brain bioenergetics [142,143]. The greater risk of developing Alzheimer’s disease in women than in men is explained by a sharp decrease in the level of sex hormones in menopausal women and therefore greater age-related atrophy in the brain structures important in the pathogenesis of this disease. Some data have indicated that older age at menopause and a longer reproductive period in women are associated with a lower risk of depression later in life and may also be associated with delayed cognitive decline [144,145]. However, in contrast to the well-defined neuroprotective and enhancing cognitive function effects of 17ß-estradiol in preclinical studies, the action of hormone replacement therapy (HRT) on age-related cognitive decline is ambiguous. The beneficial effect of estrogen supplementation is recognized in women in some studies, but these results have not been confirmed by international clinical trials [137,146]. The differences in the effects of hormone replacement therapy in women are probably due to the time of HRT introduction (better effect with an earlier start of treatment), administration of various estrogenic and progestogenic compounds (17ß-estradiol more effective than estrone; progesterone has a better effect than synthetic medroxyprogesterone acetate), route of administration (oral vs. transdermal) and occurrence of other risk factors (e.g., apolipoprotein E, mtDNA haplogroup).

Data from animal studies indicate that loss of ovarian hormones decreases brain mitochondrial function, including the process of oxidative phosphorylation, ATP production and expression or activity of some mitochondrial metabolic enzymes. For example, it has been shown that chronic ovariectomy reduces oxygen consumption, ATP production rates and mitochondrial membrane potential in NADH-associated respiration in Wistar adult female rats’ hippocampal mitochondria [147]. These data suggest that the lack of ovarian hormones leads to a decrease in the function of mainly complex I in the respiratory chain. Moreover, these authors also showed that ovarian hormone deprivation causes changes in the mitochondrial membrane lipid profile, including lowering of the level of cardiolipin, a glycerolophospholipid that reduces the permeability of the mitochondrial membrane and increases the stability of complexes and supercomplexes, and thus the energy efficiency of cellular respiration [147,148]. Cardiolipin, the level of which falls with age, is needed to maintain cytochrome c function, and its weaker action may disturb the physiological intensification of oxidative phosphorylation under stress conditions. Accordingly, it was found that exposure of neurons taken from old rats to 17β-estradiol increases cardiolipin levels and enhances mitochondrial respiration under glutamate stress [148].

The beneficial effects of hormone replacement therapy on cognitive function in postmenopausal women are mainly attributed to 17ß-estradiol action, but estrogen is usually given together with progesterone derivative, and although the role of progesterone is less defined, this hormone also exerts a neuroprotective effect under certain conditions. Progesterone regulates neuronal development of Purkinje cells in the cerebellum, differentiation and proliferation of oligodendrocytes, synaptogenesis, and neuronal plasticity, promotes myelin production and modulates neuroinflammation. Studies conducted in adrenalectomized Sprague–Dawley rats have shown that administration of progesterone, such as 17ß-estradiol, significantly increases brain mitochondrial respiration, activity and expression of complex IV and expression of complex V but does not affect mitochondrial biogenesis [149]. Interestingly, in this study, both progesterone and E_2_ reduced the leakage of free radicals, indicating a greater efficiency of electron transport, and in accordance with this effect, they reduced mitochondrial lipid peroxidation. Even though both of these hormones increased oxidative phosphorylation and decreased leakage of free radicals and lipid peroxidation and, on some parameters, even more strongly acted progesterone, after the combined administration of progesterone and 17ß-estradiol, no increase in estrogen action but even a decrease in some metabolic effects was observed [149]. These data suggest that these two steroids may act via two different, overlapping mechanisms between which there may be synergism or antagonism. In contrast to the attenuation of estradiol-induced metabolic changes by progesterone, the effect on the antioxidant system was preserved. This phenomenon can to a certain extent explain the various effects of hormone replacement therapy observed in postmenopausal women. However, in the case of HRT, the type of progestin administered may be more important because in the case of medroxyprogesterone acetate (MPA), a compound most used in contraception and hormone therapies, no attenuation but suppression of the effect of estrogen was observed [150]. In addition, MPA inhibited not only the beneficial effect of 17ß-estradiol on the ovariectomy-induced decrease in mitochondrial bioenergetic function but also the coadministration of MPA, which was detrimental to the antioxidant defense system. These results indicate significant differences between the effects of progesterone and MPA on oxidative phosphorylation, the level/activity of antioxidant enzymes and lipid peroxidation [150]. Moreover, apart from the differences in the effects of progesterone and MPA, various effects of progesterone depending on its administration were also observed. More beneficial action on genes involved in mitochondrial bioenergetics, redox homeostasis and insulin signaling was observed after more physiological, cyclic progesterone administration than after chronic, continuous exposure to this steroid [151].

The beneficial effects of both 17ß-estradiol and progesterone, but not MPA, on antioxidant defense are particularly important because the brain, due to weak antioxidant activity and strong production of free radicals during mitochondrial respiration, and the metabolism of some neurotransmitters, mainly dopamine, is particularly sensitive to oxidative stress-induced damage. Oxidative stress and the related induction of apoptosis are believed to be the main mechanisms responsible for neuronal damage both in age and in neurodegenerative diseases (Alzheimer’s disease, Parkinson’s disease) and brain damage (ischemic stroke, traumatic brain injury). It is also known that some unfavorable changes in CNS cells may begin many years earlier than clinical symptoms of neurodegenerative diseases appear, and presumably the first disturbances concern mitochondrial dysfunction, which may induce oxidative stress followed by apoptosis.

### 5.2. The Influence of Androgens on Oxidative Phosphorylation

The neuroprotective and cognitive effects of testosterone, although less studied than those of 17ß-estradiol and progesterone, have also been demonstrated. For example, it has been found that testosterone and its active metabolite dihydrotestosterone reduce neurological deficits and brain damage in a focal cerebral ischemia model and weaken astrogliosis and microgliosis caused by traumatic brain injury [152,153]. On the other hand, lowering testosterone levels increases the susceptibility of the cerebral cortex and the hippocampus to damage caused by oxidative stress [154]. The protective effect of this steroid is associated mainly with its antioxidant activity, decreasing mitochondrial apoptosis and intensifying brain-derived neurotrophic factor (BDNF) production [155]. Currently, increasing data indicate that in terms of the neuroprotective effect of testosterone, especially protecting against damage induced by oxidative stress, its influence on the improvement of mitochondrial function plays an important role. Male gonadectomy (GDX) rats have been shown to have decreased expression of genes encoding mitochondrial respiratory chain proteins [156]. Moreover, the expression of the major regulator of mitochondrial biogenesis, PGC-1α, and its downstream transcription factors were also decreased. Interestingly, these changes occurred in the hippocampus and not in the cortex, which suggests that lowering testosterone levels (for example, age-related) may be the cause of hippocampus-dependent memory deficits. Regarding the mechanism underlying the protective effect of testosterone on the function of mitochondria, its influence on the level of neuroglobin also plays an important role [157]. Testosterone increases the synthesis of neuroglobin, an oxygen sensor protein involved in its transport and ROS scavenging, interacts with cytochrome c in mitochondria and inhibits its translocation to the cytoplasm and, as a result, the activation of apoptosis.

### 5.3. The Role of Sex Steroids in Brain Damage—Gender Differences in Brain Cell Damage Mechanisms

Current research indicates that impaired mitochondrial function plays a major role not only in brain aging but also in the pathogenesis of some neurodegenerative diseases and changes induced by brain damage [158]. In line with this concept, the beneficial effect of ovarian hormones on brain mitochondrial function seems to be responsible for better protection of females in the reproductive period than males after cerebral ischemia. Mitochondria, which regulate energy production, oxidative stress, calcium homeostasis and the apoptotic process, play an important role in the damage to nerve cells caused by ischemia-reperfusion. Sex-related differences in both the severity of damage and the mechanism of inducing cell death in stroke have been demonstrated. In males, cell death is mainly connected with activation of poly(ADP ribose) polymerase 1 (PARP-1), whereas in females, primary death is evoked by caspase activation. Accordingly, it has been shown that a decrease in the size of the brain infarct is caused by PARP-1 and NO synthase inhibition in males and by caspase inhibitors in females [159,160]. Moreover, other changes in mitochondrial function were observed in male and female mice 6 h after middle cerebral artery occlusion (MCAO). A decrease in oxygen consumption in the presence of succinate (FADH_2_-linked respiration) and a decrease in complex II activity were observed only in females, while oxygen consumption in the presence of pyruvate (NADH-linked respiration) was lowered more in males than in females [137]. Since ATP synthesis depends on NADH-linked respiration, this suggests that a greater decrease in ATP levels may be induced by MCAO in males than in females, which would explain the stronger endogenous protection in females. In the MCAO-induced injury model, some data indicate the protective effect of progesterone, which, when administered 1 h after ischemia, normalized mitochondrial respiration and reduced damage in males and females, whereas some studies show a beneficial effect of progesterone in males only [161,162].

Low levels of testosterone in men have also been shown in certain neurodegenerative diseases, such as Alzheimer’s disease, Parkinson’s disease, vascular dementia, amyotrophic lateral sclerosis, and Huntington’s disease [163]. The protective effect of androgens in Alzheimer’s disease results not only from reducing the susceptibility of neurons to apoptosis but also from increasing the expression of the Aβ-catabolizing enzyme neprilysin and decreasing Aβ accumulation [163].

### 5.4. Mitochondrial Dynamics and Neurosteroid Synthesis

**Figure 3:**
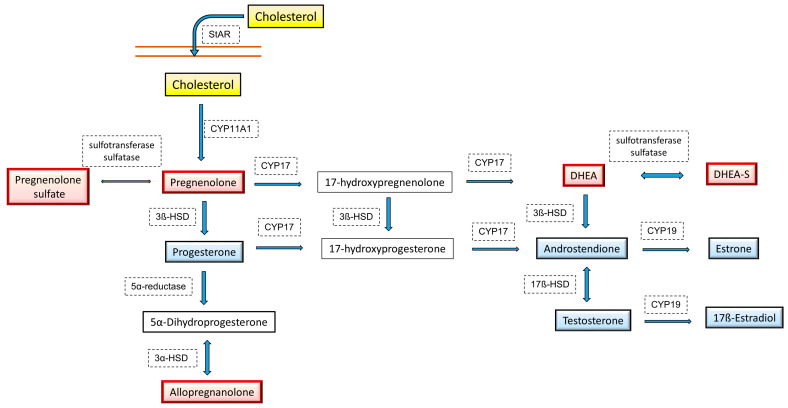
A simplified diagram of the synthesis of steroid hormones and neurosteroids. DHEA—dehydroepiandrosterone; DHEA-S—dehydroepiandrosterone sulfate; CYP11A1—mitochondrial cholesterol side-chain cleavage enzyme; StAR—steroidogenic acute-regulatory protein; 3β-HSD—3β-hydroxysteroid dehydrogenase; CYP17-hydroxylase/17,20-lyase; CYP19—aromatase; 17β-HSD—17β-hydroxysteroid dehydrogenases; 3α-HSD—3α-hydroxysteroid oxidoreductase. Sex steroids are presented in blue boxes whereas neurosteroids are featured in red boxes.

The decrease in the levels of sex hormones with age, much stronger in women than in men, may be a significant cause of the reduction in mitochondrial function, but mitochondrial dysfunction may also reduce the synthesis of sex steroids. The level of sex hormones in the brain does not fully correlate with their concentration in the blood because they can also be synthesized locally, and mitochondria are the site of the first step of steroidogenesis. The precursor to all steroid hormones and neurosteroids is cholesterol, which is transferred from the outer to inner mitochondrial membrane by the steroidogenic acute regulatory (StAR) protein and then converted to pregnenolone by cytochrome P450 side-chain cleavage (P450scc; CYP11A1) enzyme (Figure 3).

StAR and transport protein (TSPO) are the main components mediating the transport of cholesterol into the mitochondria. Current research indicates that the synthesis of pregnenolone, a precursor to all other steroids, is regulated by the circadian clock, which in turn depends on diurnal changes in mitochondrial dynamics [164]. Mitochondrial fusion correlates with pregnenolone production, and disturbance of the mechanism regulating the circadian cycle disturbs both the rhythm of changes in the dynamics of mitochondria and the synthesis of pregnenolone. Thus, circadian regulation of mitochondrial fusion-fission processes affects not only the process of oxidative phosphorylation and ATP production but also steroidogenesis. Moreover, it is believed that the neuroprotective effects of TSPO ligands in Alzheimer’s disease, compounds that increase mitochondrial respiration and decrease ROS production, may be due to enhanced neurosteroid synthesis [165]. In addition to pregnenolone, other steroids can also attenuate the mitochondrial disturbances seen in cellular models of Alzheimer’s disease. Thus, a disturbance in the dynamics of the mitochondria may weaken the process of steroidogenesis in the brain, and decreased levels of neurosteroids and sex hormones can further aggravate mitochondrial dysfunction. A similar relationship between the dynamics of mitochondria and the synthesis of steroid hormones was also demonstrated in the periphery. For example, a correlation between mitochondrial fusion and progesterone synthesis in testicular Leydig cells was found, and both processes were downregulated in the case of mitofusin 2 deficiency, a protein involved in the fusion of outer mitochondrial membranes [166].

### 5.5. The Role of Neurosteroids in the Regulation of Mitochondrial Function

**Figure 4:**
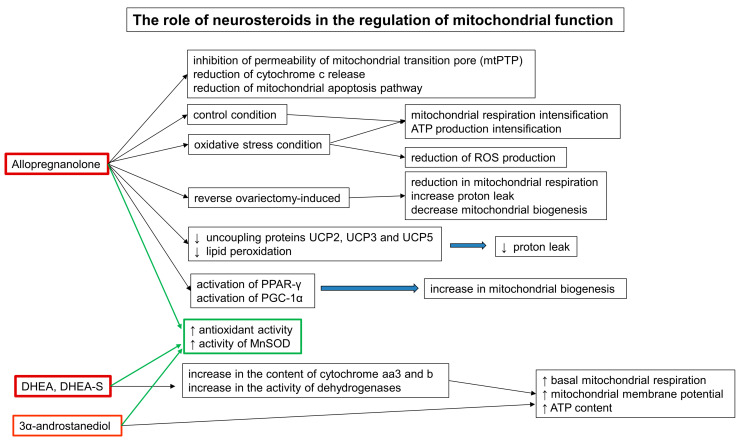
A schematic diagram illustrating the effects of neurosteroids on the regulation of mitochondrial function. DHEA—dehydroepiandrosterone; DHEA-S—dehydroepiandrosterone sulfate; 3α-androstanediol—5α-androstano-3α, 17β-diol; PGC-1α—peroxisome proliferator-activated receptor-γ coactivator 1α; PPAR-γ—peroxisome proliferator-activated receptor γ; MnSOD—manganese superoxide dismutase.

Classic steroid hormones act in the brain as well as in the periphery, mainly through intracellular nuclear hormone receptors, while neurosteroids generally act on membrane receptors, mainly GABA_A_, NMDA, and Sigma 1. Neurosteroids are synthesized in the brain and remain in the brain even after peripheral endocrine glands are removed [167]. By regulating neurotransmission, they influence many functions of the CNS, and most of them have been shown to have neuroprotective effects. The greatest amount of data on neuroprotective effects concerns allopregnanolone (3α,5α-tetrahydroprogesterone), a metabolite of progesterone. Both allopregnanolone and progesterone have beneficial effects on the damage caused by various factors to nerve cells, and current research indicates that enhancing mitochondrial bioenergetics may be the main mechanism underlying their neuroprotective effects (Figure 4). In models of traumatic and ischemic brain injury, administration of allopregnanolone, stronger than progesterone, inhibits the permeability of the mtPTP, a key element in the induction of the mitochondrial apoptosis pathway and decreases cytochrome c release [168]. It was found that allopregnanolone and its agonists in control cultures of SH-SY5Y cells and amyloid-beta precursor protein (APP)/Aβ-overexpressing cells, which are an in vitro model of Alzheimer’s disease, increase ATP synthesis and intensify mitochondrial respiration under baseline conditions and in the presence of oxidative stress [169]. In addition, under oxidative stress, allopregnanolone and its agonist BR 297 also decreased ROS production and prevented cell death. This study further suggests that the mitochondrial respiration-enhancing and ROS-depleting effects of allopregnanolone are not due to its best-known activity as a positive allosteric GABA_A_ receptor modulator but rather an effect on intracellular pathways that control oxidative phosphorylation. The fact that the stable analog of allopregnanolone, the compound BR 297, with a weak effect on the GABA_A_ receptor, exerts a stronger, beneficial effect on mitochondrial function and ROS production than allopregnanolone, and additionally only it increases the capacity of substrate oxidation when cells have high energy demands, indicating the existence of another mechanism of action of these compounds on mitochondrial bioenergetics and cell survival, other than through the GABA receptor. The reversal of bioenergy deficits by allopregnanolone has also been demonstrated in vivo in an animal model of Alzheimer’s disease. Administration of allopregnanolone has been found to reverse ovariectomy-induced reduction in mitochondrial respiration, increase proton leak, and decrease mitochondrial biogenesis [170]. When examining the mechanism of action of allopregnanolone, it was found that the reduction in proton leakage may be due to the reduction in the expression of the uncoupling proteins UCP2, UCP3 and UCP5, as well as the inhibition of lipid peroxidation, whereas the increase in mitochondrial biogenesis may be due to activation of key modulators of this process, that is, peroxisome proliferator-activated receptor-γ and peroxisome proliferator-activated receptor-γ coactivator 1α. Moreover, allopregnanolone enhances glucose metabolism by enhancing the expression or activity of pyruvate dehydrogenase and α-ketoglutarate dehydrogenase. The second essential neurosteroid that can be synthesized in the brain is dehydroepiandrosterone (DHEA) and its sulfated conjugate DHEA-S. DHEA regulates many functions of the brain, including neuronal plasticity, neuronal survival, and cognition, and its reduced level seems to be involved in the pathogenesis of depression and neurodegenerative diseases, including Alzheimer’s disease. DHEA administration increased oxidative phosphorylation and ATPase activity in the mitochondria of developing rats [171]. Additionally, administration of DHEA to adult male rats significantly stimulates oxidative phosphorylation in the brain’s mitochondria and increases the production of ATP [172]. The intensification of mitochondrial respiration by DHEA is accompanied by an increase in the content of cytochrome aa3 and b and an increase in the activity of dehydrogenases (Figure 4).

In light of current research, it seems that the ability to intensify the function of mitochondria in the brain is demonstrated not only by the abovementioned hormones, such as 17ß-estradiol, progesterone, testosterone and neurosteroids such as allopregnanolone and DHEA, but also by the common mechanism of action of many steroids in the brain. In primary neuronal cultures, in addition to the steroids mentioned above, compounds such as estrone and 3α-androstanediol are able to improve bioenergetic activity by increasing basal mitochondrial respiration, mitochondrial membrane potential and ATP content [173]. An important aspect of their action is the fact that by increasing oxidative phosphorylation, which often leads to intensification of the production of mitochondrial reactive oxygen species (mtROS), they simultaneously heighten antioxidant activity, mainly by enhancing the activity of manganese superoxide dismutase activity (MnSOD), an enzyme located in the mitochondrial matrix.

Since the synthesis of sex steroids and neurosteroids decreases with age as well as in neurodegenerative diseases, in parallel with the occurrence of mitochondrial dysfunction and the weakening of cognitive functions, steroids through their pleiotropic effects, especially the intensification of oxidative phosphorylation, may find wider clinical application in the future, both in terms of delaying the aging process and the treatment of certain neurodegenerative diseases.

It is apparent that CNS cells can be both the target and also the source of many hormones, the question arises whether the hormones synthesized in the brain may have a different effect on its functioning, including bioenergetic processes and mitochondrial function when compared to hormones which reach to the brain from the periphery. Unfortunately, the data on possible differences in effects between hormones synthesized in the brain and derived from the periphery are very scarce, although such differences have been observed several times in the case of steroid hormones. For example, Chen et al. [174] demonstrate that hippocampal E_2_, but not peripheral E_2_, influences cognitive functions assessed in the novel object recognition test in female rats. In addition, studies, which found that the neuroprotective effect of E_2_ in brain damage depends on the intensity of aromatase expression in reactive astrocytes, indicate the role of locally synthesized estradiol in promoting the viability of neurons [175]. Previous studies comparing mitochondrial respiration in the brain between female and male control and gonadectomy mice showed that females have higher a mitochondrial respiration and lower oxidative stress compared to males and that these differences were suppressed by ovariectomy. which proves the role of peripheral steroids [136]. In addition, the fact that chronic ovarian hormone deprivation induces mitochondrial dysfunction in the brain, which may contribute to an increased risk of Alzheimer’s disease and other age-related disorders seen in postmenopausal women, also demonstrates the protective effect of peripheral hormones [147]. Thus, it is currently not possible to determine the role of steroids synthesized locally in the brain as compared to those derived from the periphery and there is no existing data, which may distinguish their influence on mitochondrial function.

## 6. Conclusions

Research conducted over recent decades has significantly changed our view of the effects of hormones in the body by extending knowledge about the role of endocrine signaling in the complex interactions between the body and the brain. It is currently clearly evident that neurons and glia respond to peripheral hormone signaling, and moreover, some peptide hormones, such as insulin, IGF-1 or GLP-1, can also be synthetized locally in particular regions of the brain or, as in the case of neurosteroids, are synthesized mainly in brain cells. Proper hormonal signaling is important for the regulation of neuronal survival, synaptic plasticity, neurogenesis, neurotransmission and cognitive function, and many of their effects may be mediated by the modulation of energy metabolism since the action of different hormones in the brain on the one hand was shown to affect the production of ATP and the activity and protein expression of mitochondrial respiratory complexes. On the other hand, hormone-mediated signaling was shown to influence many processes related to the structural and functional stability of the mitochondrial network, such as their biogenesis, fusion, fission, stress response, calcium homeostasis and redox balance.

Deficiencies in the action of many hormones, including insulin and thyroid hormones, estrogens and androgens, can impair brain functions, including mood, memory and cognition, and moreover, their decreased action in the long run can promote neurodegenerative changes, thereby significantly contributing to the development of CNS diseases, particularly dementias and depressive symptoms. Although the mechanisms by which disturbances in central hormone signaling contribute to the development of neurodegenerative changes are complex, increased action of free radicals linked to impaired OXPHOS efficacy and stability of the mitochondrial network has repeatedly been shown to play a role.

Different hormone-based therapies, which are implemented to counteract deficiencies in endogenous hormone signaling, have been proven to exert numerous beneficial effects on brain function in patients, which in some cases include the improvement of brain energy metabolism. Depending on a particular hormone, targeting respiratory processes in mitochondria may be achieved indirectly by activating certain intracellular pathways upon the binding of hormone to its specific receptor, as is most likely achieved by peptide hormones; however, it was also found that in the case of steroid hormones such as glucocorticoids, modulation of mitochondrial function is also achieved directly by activating mitochondrial GR receptors. At this point, in the context of physiological outcomes of hormonal action, glucocorticoids are a good example to illustrate the differences between short- and long-term hormone action. Glucocorticoids are necessary to maintain proper brain function during stressful events; however, their prolonged excessive action results in pathological brain changes, which also include impairments in mitochondrial function and increased oxidative stress.

In this review, we provided evidence that the regulation of energy metabolism in the brain is an important aspect of the central action of many hormones, and we also provided examples showing that both insufficient and, in some cases, excessive hormone action may contribute to the formation of pathological changes in the brain. It is worth noting, however, that despite the existence of numerous experimental data, the intracellular mechanisms related to the influence of hormones on particular aspects related to the regulation of mitochondrial OXPHOS are still not sufficiently understood. Therefore, more detailed studies aimed at understanding the molecular regulatory mechanisms of metabolic processes by individual hormones are needed to not only better understand the complex role of endocrine signaling in the brain under both physiological and pathophysiological conditions but also provide a valuable foundation for the possibility of using hormone therapies in the treatment of central nervous system diseases, such as cognitive impairment and depression.

## References

[1] Malcomson R.D.G., Nagy A. (2015). The Endocrine System.

[2] Clarke I. (2014). Hypothalamus as an Endocrine Organ. Compr. Physiol..

[3] McEwen B.S. (2020). Hormones and behavior and the integration of brain-body science. Horm. Behav..

[4] Biessels G.J., Reagan L.P. (2015). Hippocampal insulin resistance and cognitive dysfunction. Nat. Rev. Neurosci..

[5] Sheline Y.I., Liston C., McEwen B.S. (2019). Parsing the Hippocampus in Depression: Chronic Stress, Hippocampal Volume, and Major Depressive Disorder. Biol. Psychiatry.

[6] Macdonald R., Barnes K., Hastings C., Mortiboys H. (2018). Mitochondrial abnormalities in Parkinson’s disease and Alzheimer’s disease: Can mitochondria be targeted therapeutically?. Biochem. Soc. Trans..

[7] Allen J., Romay-Tallon R., Brymer K.J., Caruncho H.J., Kalynchuk L.E. (2018). Mitochondria and Mood: Mitochondrial Dysfunction as a Key Player in the Manifestation of Depression. Front. Neurosci..

[8] Roberts R.C. (2017). Postmortem studies on mitochondria in schizophrenia. Schizophr. Res..

[9] Pei L., Wallace D.C. (2018). Mitochondrial Etiology of Neuropsychiatric Disorders. Biol. Psychiatry.

[10] Jardim F.R., de Rossi F.T., Nascimento M.X., Barros R.G.D.S., Borges P.A., Prescilio I.C., De Oliveira M.R. (2017). Resveratrol and Brain Mitochondria: A Review. Mol. Neurobiol..

[11] Zhao Q., Wang J., Levichkin I.V., Stasinopoulos S., Ryan M., Hoogenraad N.J. (2002). A mitochondrial specific stress response in mammalian cells. EMBO J..

[12] Wai T., Langer T. (2016). Mitochondrial Dynamics and Metabolic Regulation. Trends Endocrinol. Metab..

[13] El-Hattab A.W., Suleiman J., Almannai M., Scaglia F. (2018). Mitochondrial dynamics: Biological roles, molecular machinery, and related diseases. Mol. Genet. Metab..

[14] Pellerin L., Magistretti P.J. (2003). Food for Thought: Challenging the Dogmas. Br. J. Pharmacol..

[15] Bélanger M., Allaman I., Magistretti P.J. (2011). Brain Energy Metabolism: Focus on Astrocyte-Neuron Metabolic Cooperation. Cell Metab..

[16] Picard M., McEwen B.S. (2018). Psychological Stress and Mitochondria: A Conceptual Framework. Psychosom. Med..

[17] Röder P.V., Wu B., Liu Y., Han W. (2016). Pancreatic regulation of glucose homeostasis. Exp. Mol. Med..

[18] Gray S.M., Meijer R.I., Barrett E.J. (2014). Insulin Regulates Brain Function, but How Does It Get There?. Diabetes.

[19] Vogt M.C., Brüning J.C. (2013). CNS insulin signaling in the control of energy homeostasis and glucose metabolism—From embryo to old age. Trends Endocrinol. Metab..

[20] Blazquez E., Velã¡zquez E., Hurtado-Carneiro V., Ruiz-Albusac J.M. (2014). Insulin in the Brain: Its Pathophysiological Implications for States Related with Central Insulin Resistance, Type 2 Diabetes and Alzheimer’sDisease. Front. Endocrinol..

[21] Garcia-Segura L., Pérez J., Pons S., Rejas M., Aleman I.T. (1991). Localization of insulin-like growth factor I (IGF-I)-like immunoreactivity in the developing and adult rat brain. Brain Res..

[22] Russo V.C., Gluckman P.D., Feldman E., Werther G.A. (2005). The Insulin-Like Growth Factor System and Its Pleiotropic Functions in Brain. Endocr. Rev..

[23] Kleinridders A. (2016). Deciphering Brain Insulin Receptor and Insulin-Like Growth Factor 1 Receptor Signalling. J. Neuroendocr..

[24] Leonard B.E., Wegener G. (2019). Inflammation, insulin resistance and neuroprogression in depression. Acta Neuropsychiatr..

[25] Shieh J.C.-C., Huang P.-T., Lin Y.-F. (2020). Alzheimer’s Disease and Diabetes: Insulin Signaling as the Bridge Linking Two Pathologies. Mol. Neurobiol..

[26] Maciejczyk M., Żebrowska E., Chabowski A. (2019). Insulin Resistance and Oxidative Stress in the Brain: What’s New?. Int. J. Mol. Sci..

[27] Mastrocola R., Restivo F., Vercellinatto I., Danni O., Brignardello E., Aragno M., Boccuzzi G. (2005). Oxidative and nitrosative stress in brain mitochondria of diabetic rats. J. Endocrinol..

[28] Logan S., Pharaoh G.A., Marlin M.C., Masser D.R., Matsuzaki S., Wronowski B., Yeganeh A., Parks E.E., Premkumar P., Farley J.A. (2018). Insulin-like growth factor receptor signaling regulates working memory, mitochondrial metabolism, and amyloid-β uptake in astrocytes. Mol. Metab..

[29] Pharaoh G., Owen D., Yeganeh A., Premkumar P., Farley J., Bhaskaran S., Ashpole N., Kinter M., Van Remmen H., Logan S. (2019). Disparate Central and Peripheral Effects of Circulating IGF-1 Deficiency on Tissue Mitochondrial Function. Mol. Neurobiol..

[30] Wardelmann K., Blümel S., Rath M., Alfine E., Chudoba C., Schell M., Cai W., Hauffe R., Warnke K., Flore T. (2019). Insulin action in the brain regulates mitochondrial stress responses and reduces diet-induced weight gain. Mol. Metab..

[31] Ruegsegger G.N., Vanderboom P.M., Dasari S., Klaus K.A., Kabiraj P., McCarthy C.B., Lucchinetti C.F., Nair K.S. (2019). Exercise and metformin counteract altered mitochondrial function in the insulin-resistant brain. JCI Insight.

[32] Schell M., Wardelmann K., Kleinridders A. (2021). Untangling the effect of insulin action on brain mitochondria and metabolism. J. Neuroendocr..

[33] Chen Y., Dai C.-L., Wu Z., Iqbal K., Liu F., Zhang B., Gong C.-X. (2017). Intranasal Insulin Prevents Anesthesia-Induced Cognitive Impairment and Chronic Neurobehavioral Changes. Front. Aging Neurosci..

[34] Guo Z., Chen Y., Mao Y.-F., Zheng T., Jiang Y., Yan Y., Yin X., Zhang B. (2017). Long-term treatment with intranasal insulin ameliorates cognitive impairment, tau hyperphosphorylation, and microglial activation in a streptozotocin-induced Alzheimer’s rat model. Sci. Rep..

[35] Benedict C. (2004). Intranasal insulin improves memory in humans. Psychoneuroendocrinology.

[36] Hallschmid M., Benedict C., Schultes B., Fehm H.-L., Born J., Kern W. (2004). Intranasal Insulin Reduces Body Fat in Men but not in Women. Diabetes.

[37] Iravanpour F., Dargahi L., Rezaei M., Haghani M., Heidari R., Valian N., Ahmadiani A. (2021). Intranasal insulin improves mitochondrial function and attenuates motor deficits in a rat 6-OHDA model of Parkinson’s disease. CNS Neurosci. Ther..

[38] Torabi N., Noursadeghi E., Shayanfar F., Nazari M., Fahanik-Babaei J., Saghiri R., Khodagholi F., Eliassi A. (2021). Intranasal insulin improves the structure–function of the brain mitochondrial ATP–sensitive Ca2+ activated potassium channel and respiratory chain activities under diabetic conditions. Biochim. Et Biophys. Acta (BBA)-Mol. Basis Dis..

[39] Jauch-Chara K., Friedrich A., Rezmer M., Melchert U.H., Scholand-Engler H.G., Hallschmid M., Oltmanns K.M. (2012). Intranasal Insulin Suppresses Food Intake via Enhancement of Brain Energy Levels in Humans. Diabetes.

[40] Owen M.R., Doran E., Halestrap A.P. (2000). Evidence that metformin exerts its anti-diabetic effects through inhibition of complex 1 of the mitochondrial respiratory chain. Biochem. J..

[41] Portela L.V., Gnoatto J., Brochier A., Haas C.B., de Assis A., De Carvalho A.K., Hansel G., Zimmer E.R., Oses J.P., Muller A.P. (2014). Intracerebroventricular Metformin Decreases Body Weight But Has Pro-oxidant Effects and Decreases Survival. Neurochem. Res..

[42] Skemiene K., Rekuviene E., Jekabsone A., Cizas P., Morkuniene R., Borutaite V. (2020). Comparison of Effects of Metformin, Phenformin, and Inhibitors of Mitochondrial Complex I on Mitochondrial Permeability Transition and Ischemic Brain Injury. Biomolecules.

[43] Pintana H., Apaijai N., Pratchayasakul W., Chattipakorn N., Chattipakorn S.C. (2012). Effects of metformin on learning and memory behaviors and brain mitochondrial functions in high fat diet induced insulin resistant rats. Life Sci..

[44] Pipatpiboon N., Pratchayasakul W., Chattipakorn N., Chattipakorn S.C. (2012). PPARγ Agonist Improves Neuronal Insulin Receptor Function in Hippocampus and Brain Mitochondria Function in Rats with Insulin Resistance Induced by Long Term High-Fat Diets. Endocrinology.

[45] Sa-Nguanmoo P., Tanajak P., Kerdphoo S., Jaiwongkam T., Pratchayasakul W., Chattipakorn N., Chattipakorn S.C. (2017). SGLT2-inhibitor and DPP-4 inhibitor improve brain function via attenuating mitochondrial dysfunction, insulin resistance, inflammation, and apoptosis in HFD-induced obese rats. Toxicol. Appl. Pharmacol..

[46] Müller T., Finan B., Bloom S., D’Alessio D., Drucker D., Flatt P., Fritsche A., Gribble F., Grill H., Habener J. (2019). Glucagon-like peptide 1 (GLP-1). Mol. Metab..

[47] Kastin A.J., Akerstrom V., Pan W. (2002). Interactions of Glucagon-Like Peptide-1 (GLP-1) with the Blood-Brain Barrier. J. Mol. Neurosci..

[48] Yamamoto H., Kishi T., Lee C.E., Choi B.J., Fang H., Hollenberg A.N., Drucker D.J., Elmquist J.K. (2003). Glucagon-Like Peptide-1-Responsive Catecholamine Neurons in the Area Postrema Link Peripheral Glucagon-Like Peptide-1 with Central Autonomic Control Sites. J. Neurosci..

[49] Daniels D., Mietlicki-Baase E.G. (2018). Glucagon-Like Peptide 1 in the Brain: Where Is It Coming From, Where Is It Going?. Diabetes.

[50] Turton M.D., O’Shea D., Gunn I., Beak S.A., Edwards C.M.B., Meeran K., Choi S.J., Taylor G.M., Heath M.M., Lambert P.D. (1996). A role for glucagon-like peptide-1 in the central regulation of feeding. Nature.

[51] Kanoski S.E., Hayes M.R., Skibicka K.P. (2016). GLP-1 and weight loss: Unraveling the diverse neural circuitry. Am. J. Physiol. Integr. Comp. Physiol..

[52] Brierley D.I., Holt M.K., Singh A., de Araujo A., McDougle M., Vergara M., Afaghani M.H., Lee S.J., Scott K., Maske C. (2021). Central and peripheral GLP-1 systems independently suppress eating. Nat. Metab..

[53] Diz-Chaves Y., Herrera-Pérez S., González-Matías L.C., Lamas J.A., Mallo F. (2020). Glucagon-Like Peptide-1 (GLP-1) in the Integration of Neural and Endocrine Responses to Stress. Nutrients.

[54] Heppner K.M., Kirigiti M., Secher A., Paulsen S.J., Buckingham R., Pyke C., Knudsen L.B., Vrang N., Grove K.L. (2015). Expression and distribution of glucagon-like peptide-1 receptor mRNA, protein and binding in the male nonhuman primate (Macaca mulatta) brain. Endocrinology.

[55] Spielman L.J., Gibson D.L., Klegeris A. (2017). Incretin hormones regulate microglia oxidative stress, survival and expression of trophic factors. Eur. J. Cell Biol..

[56] Timper K., del Río-Martín A., Cremer A.L., Bremser S., Alber J., Giavalisco P., Varela L., Heilinger C., Nolte H., Trifunovic A. (2020). GLP-1 Receptor Signaling in Astrocytes Regulates Fatty Acid Oxidation, Mitochondrial Integrity, and Function. Cell Metab..

[57] Hamilton A., Patterson S., Porter D., Gault V., Holscher C. (2011). Novel GLP-1 mimetics developed to treat type 2 diabetes promote progenitor cell proliferation in the brain. J. Neurosci. Res..

[58] Athauda D., Foltynie T. (2016). The glucagon-like peptide 1 (GLP) receptor as a therapeutic target in Parkinson’s disease: Mechanisms of action. Drug Discov. Today.

[59] Kim Y.-K., Kim O.Y., Song J. (2020). Alleviation of Depression by Glucagon-Like Peptide 1 through the Regulation of Neuroinflammation, Neurotransmitters, Neurogenesis, and Synaptic Function. Front. Pharmacol..

[60] Kastin A.J., Akerstrom V. (2003). Entry of exendin-4 into brain is rapid but may be limited at high doses. Int. J. Obes..

[61] Hunter K., Hölscher C. (2012). Drugs developed to treat diabetes, liraglutide and lixisenatide, cross the blood brain barrier and enhance neurogenesis. BMC Neurosci..

[62] Pintana H., Apaijai N., Chattipakorn N., Chattipakorn S.C. (2013). DPP-4 inhibitors improve cognition and brain mitochondrial function of insulin-resistant rats. J. Endocrinol..

[63] He W., Wang H., Zhao C., Tian X., Li L., Wang H. (2019). Role of liraglutide in brain repair promotion through Sirt1-mediated mitochondrial improvement in stroke. J. Cell. Physiol..

[64] Xie Y., Zheng J., Li S., Li H., Zhou Y., Zheng W., Zhang M., Liu L., Chen Z. (2021). GLP-1 improves the neuronal supportive ability of astrocytes in Alzheimer’s disease by regulating mitochondrial dysfunction via the cAMP/PKA pathway. Biochem. Pharmacol..

[65] Zheng J., Xie Y., Ren L., Qi L., Wu L., Pan X., Zhou J., Chen Z., Liu L. (2021). GLP-1 improves the supportive ability of astrocytes to neurons by promoting aerobic glycolysis in Alzheimer’s disease. Mol. Metab..

[66] Shupnik M.A., Ridgway E.C., Chin W.W. (1989). Molecular Biology of Thyrotropin. Endocr. Rev..

[67] Taylor P.N., Albrecht D., Scholz A., Gutierrez-Buey G., Lazarus J.H., Dayan C.M., Okosieme O.E. (2018). Global epidemiology of hyperthyroidism and hypothyroidism. Nat. Rev. Endocrinol..

[68] Emorte B., Bernal J. (2014). Thyroid Hormone Action: Astrocyte–NeuronCommunication. Front. Endocrinol..

[69] Weiss R.E., Refetoff S. (1996). Effect of thyroid hormone on growth. Endocrinol. Metab. Clin. North Am..

[70] Oppenheimer J.H. (1997). Molecular Basis of Thyroid Hormone-Dependent Brain Development. Endocr. Rev..

[71] Schroeder A.C., Privalsky M.L. (2014). Thyroid Hormones, T3 and T4, in the Brain. Front. Endocrinol..

[72] Wrutniak-Cabello C., Casas F., Cabello G. (2001). Thyroid hormone action in mitochondria. J. Mol. Endocrinol..

[73] Chaker L., Bianco A., Jonklaas J., Peeters R.P. (2017). Hypothyroidism. Lancet.

[74] De Leo S., Lee S.Y., Braverman L.E., Unit E., Sciences C. (2016). Hyperthyroidism_Lancet review. Lancet.

[75] Bauer M., Heinz A., Whybrow P.C. (2002). Thyroid hormones, serotonin and mood: Of synergy and significance in the adult brain. Mol. Psychiatry.

[76] Singh R., Upadhyay G., Godbole M.M. (2003). Hypothyroidism alters mitochondrial morphology and induces release of apoptogenic proteins during rat cerebellar development. J. Endocrinol..

[77] Fazekas J.F., Graves F.B., Alman R.W. (1951). The influence of the thyroid on cerebral metabolism. Endocrinology.

[78] Reiss J.M., Reiss M., Wyatt A. (1956). Action of Thyroid Hormones on Brain Metabolism of Newborn Rats. Exp. Biol. Med..

[79] Davies K.L., Smith D.J., El-Bacha T., Stewart M.E., Easwaran A., Wooding P.F.P., Forhead A.J., Murray A.J., Fowden A.L., Camm E.J. (2021). Development of cerebral mitochondrial respiratory function is impaired by thyroid hormone deficiency before birth in a region-specific manner. FASEB J..

[80] Martinez B., Del Hoyo P., Martin M.A., Arenas J., Perez-Castillo A., Santos A. (2001). Thyroid hormone regulates oxidative phosphorylation in the cerebral cortex and striatum of neonatal rats. J. Neurochem..

[81] Glass C.K., Rosenfeld M.G. (2000). The coregulator exchange in transcriptional functions of nuclear receptors. Genes Dev..

[82] Głombik K., Detka J., Kurek A., Budziszewska B. (2020). Impaired Brain Energy Metabolism: Involvement in Depression and Hypothyroidism. Front. Neurosci..

[83] Zhuravliova E., Barbakadze T., Jojua N., Zaalishvili E., Shanshiashvili L., Natsvlishvili N., Kalandadze I., Narmania N., Chogovadze I., Mikeladze D. (2012). Synaptic and Non-Synaptic Mitochondria in Hippocampus of Adult Rats Differ in Their Sensitivity to Hypothyroidism. Cell. Mol. Neurobiol..

[84] Vega-Núñez E., Álvarez A.M., Menéndez-Hurtado A., Santos A., Pérez-Castillo A. (1997). Neuronal Mitochondrial Morphology and Transmembrane Potential Are Severely Altered by Hypothyroidism during Rat Brain Development1. Endocrinology.

[85] Martinez B., Rodrigues T., Gine E., Kaninda J.P., Perez-Castillo A., Santos A. (2009). Hypothyroidism Decreases the Biogenesis in Free Mitochondria and Neuronal Oxygen Consumption in the Cerebral Cortex of Developing Rats. Endocrinology.

[86] Satav J.G., Katyare S.S. (1982). Effect of experimental thyrotoxicosis on oxidative phosphorylation in rat liver, kidney and brain mitochondria. Mol. Cell. Endocrinol..

[87] Schwartz H.L., Oppenheimer J.H. (1978). Ontogenesis of 3,5,3′-Triiodothyronine Receptors in Neonatal Rat Brain: Dissociation between Receptor Concentration and Stimulation of Oxygen Consumption by 3,5,3′-Triiodothyronine. Endocrinology.

[88] Silva J.E., Matthews P.S. (1984). Production rates and turnover of triiodothyronine in rat-developing cerebral cortex and cerebellum. Responses to hypothyroidism. J. Clin. Investig..

[89] Katyare S.S., Rajan R.R. (2005). Influence of thyroid hormone treatment on the respiratory activity of cerebral mitochondria from hypothyroid rats. A critical re-assessment. Exp. Neurol..

[90] Sheehan T.E., Kumar P.A., Hood D.A. (2004). Tissue-specific regulation of cytochromecoxidase subunit expression by thyroid hormone. Am. J. Physiol. Metab..

[91] Bauer M., Whybrow P.C. (2021). Role of thyroid hormone therapy in depressive disorders. J. Endocrinol. Investig..

[92] Bauer M., London E.D., Rasgon N., Berman S.M., Frye M.A., Altshuler L.L., Mandelkern M.A., Bramen J., Voytek B., Woods R. (2005). Supraphysiological doses of levothyroxine alter regional cerebral metabolism and improve mood in bipolar depression. Mol. Psychiatry.

[93] Głombik K., Detka J., Budziszewska B. (2021). Venlafaxine and L-Thyroxine Treatment Combination: Impact on Metabolic and Synaptic Plasticity Changes in an Animal Model of Coexisting Depression and Hypothyroidism. Cells.

[94] Aleksandrova L.R., Wang Y.T., Phillips A.G. (2019). Evaluation of the Wistar-Kyoto rat model of depression and the role of synaptic plasticity in depression and antidepressant response. Neurosci. Biobehav. Rev..

[95] Manoli I., Alesci S., Blackman M.R., Su Y.A., Rennert O.M., Chrousos G.P. (2007). Mitochondria as key components of the stress response. Trends Endocrinol. Metab..

[96] Vale W., Spiess J., Rivier C., Rivier J. (1982). Characterization of a 41-Residue Ovine Hypothalamic Peptide that Stimulates Secretion of Corticotropin and β-Endorphin. Obstet. Gynecol. Surv..

[97] Antoni F.A. (1986). Hypothalamic Control of Adrenocorticotropin Secretion: Advances since the Discovery of 41-Residue Corticotropin-Releasing Factor. Endocr. Rev..

[98] Gjerstad J.K., Lightman S., Spiga F. (2018). Role of glucocorticoid negative feedback in the regulation of HPA axis pulsatility. Stress.

[99] Dallman M.F., Akana S.F., Jacobson L., Levin N., Cascio C.S., Shinsako J. (1987). Characterization of Corticosterone Feedback Regulation of ACTH Secretion. Ann. N. Y. Acad. Sci..

[100] Zalachoras I., Houtman R., Meijer O. (2013). Understanding stress-effects in the brain via transcriptional signal transduction pathways. Neuroscience.

[101] McNally J.G., Muller W.G., Walker D., Wolford R., Hager G.L. (2000). The Glucocorticoid Receptor: Rapid Exchange with Regulatory Sites in Living Cells. Science.

[102] Finsterwald C., Alberini C.M. (2013). Stress and glucocorticoid receptor-dependent mechanisms in long-term memory: From adaptive responses to psychopathologies. Neurobiol. Learn. Mem..

[103] Karstens A.J., Korzun I., Avery E.T., Kassel M.T., Keelan R., Kales H., Abercrombie H., Eisenlohr-Moul T., Langenecker S.A., Weisenbach S. (2018). Examining HPA-axis functioning as a mediator of the relationship between depression and cognition across the adult lifespan. Aging, Neuropsychol. Cogn..

[104] Morin C., Zini R., Simon N., Charbonnier P., Tillement J.-P., Le Louet H. (2000). Low glucocorticoid concentrations decrease oxidative phosphorylation of isolated rat brain mitochondria: An additional effect of dexamethasone. Fundam. Clin. Pharmacol..

[105] Katyare S., Balasubramanian S., Parmar D. (2003). Effect of corticosterone treatment on mitochondrial oxidative energy metabolism in developing rat brain. Exp. Neurol..

[106] Picard M., Juster R.-P., McEwen B.S. (2014). Mitochondrial allostatic load puts the ‘gluc’ back in glucocorticoids. Nat. Rev. Endocrinol..

[107] Adzic M., Lukic I., Mitic M., Djordjevic J., Elaković I., Djordjevic A., Krstic-Demonacos M., Matić G., Radojcic M. (2013). Brain region- and sex-specific modulation of mitochondrial glucocorticoid receptor phosphorylation in fluoxetine treated stressed rats: Effects on energy metabolism. Psychoneuroendocrinology.

[108] Jaszczyk A., Juszczak G.R. (2021). Glucocorticoids, metabolism and brain activity. Neurosci. Biobehav. Rev..

[109] Scheller K., Sekeris C.E., Krohne G., Hock R., Hansen I.A., Scheer U. (2000). Localization of glucocorticoid hormone receptors in mitochondria of human cells. Eur. J. Cell Biol..

[110] Moutsatsou P., Psarra A.-M., Tsiapara A., Paraskevakou H., Davaris P., Sekeris C. (2001). Localization of the Glucocorticoid Receptor in Rat Brain Mitochondria. Arch. Biochem. Biophys..

[111] Lapp H.E., Bartlett A.A., Hunter R.G. (2019). Stress and glucocorticoid receptor regulation of mitochondrial gene expression. J. Mol. Endocrinol..

[112] Demonacos C., Karayanni N., Chatzoglou E., Tsiriyiotis C., Spandidos D., Sekeris C.E. (1996). Mitochondrial genes as sites of primary action of steroid hormones. Steroids.

[113] Tsiriyotis C., Spandidos D., Sekeris C.E. (1997). The Mitochondrion as a Primary Site of Action of Glucocorticoids: Mitochondrial Nucleotide Sequences, Showing Similarity to Hormone Response Elements, Confer Dexamethasone Inducibility to Chimaeric Genes Transfected in LATK−Cells. Biochem. Biophys. Res. Commun..

[114] Romagnoli C., Zecca E., Vento G., Maggio L., Papacci P., Tortorolo G. (1999). Effect on Growth of Two Different Dexamethasone Courses for Preterm Infants at Risk of Chronic Lung Disease. Pharmacology.

[115] Kumar P. (2005). Effect of Decreased Use of Postnatal Corticosteroids on Morbidity in Extremely Low Birthweight Infants. Am. J. Perinatol..

[116] Lorscheider M., Tsapis N., Ur-Rehman M., Gaudin F., Stolfa I., Abreu S., Mura S., Chaminade P., Espéli M., Fattal E. (2019). Dexamethasone palmitate nanoparticles: An efficient treatment for rheumatoid arthritis. J. Control. Release.

[117] Gilstrap L.C., Christensen R., Clewell W.H., D’Alton M.E., Davidson E.C., Escobedo M.B., Gjerdingen D.K., Goddard-Finegold J., Goldenberg R.L., Grimes D.A. (1995). Effect of Corticosteroids for Fetal Maturation on Perinatal Outcomes. JAMA.

[118] Neal C.R., Weidemann G., Kabbaj M., Vázquez D.M. (2004). Effect of neonatal dexamethasone exposure on growth and neurological development in the adult rat. Am. J. Physiol. Integr. Comp. Physiol..

[119] Prieur B., Bismuth J., Delaval E. (1998). Effects of adrenal steroid hormones on mitochondrial maturation during the late fetal period. JBIC J. Biol. Inorg. Chem..

[120] Holt P., Oliver I.T. (1968). Plasma corticosterone concentrations in the perinatal rat. Biochem. J..

[121] Nakai A., Shibazaki Y., Taniuchi Y., Oya A., Asakura H., Koshino T., Araki T. (2002). Effect of dexamethasone on mitochondrial maturation in the fetal rat brain. Am. J. Obstet. Gynecol..

[122] Nakai A., Taniuchi Y., Asakura H., Oya A., Yokota A., Koshino T., Araki T. (2000). Developmental changes in mitochondrial activity and energy metabolism in fetal and neonatal rat brain. Dev. Brain Res..

[123] Pandya J.D., Agarwal N.A., Katyare S.S. (2007). Dexamethasone treatment differentially affects the oxidative energy metabolism of rat brain mitochondria in developing and adult animals. Int. J. Dev. Neurosci..

[124] Sapolsky R.M., Meaney M.J. (1986). Maturation of the adrenocortical stress response: Neuroendocrine control mechanisms and the stress hyporesponsive period. Brain Res..

[125] Poyton R.O., McEwen J.E. (1996). Crosstalk between nuclear and mitochondrial genomes. Annu. Rev. Biochem..

[126] McEwen B.S. (2005). Glucocorticoids, depression, and mood disorders: Structural remodeling in the brain. Metabolism.

[127] Yu S., Holsboer F., Almeida O.F. (2008). Neuronal actions of glucocorticoids: Focus on depression. J. Steroid Biochem. Mol. Biol..

[128] Cabib S., Campus P., Conversi D., Orsini C., Puglisi-Allegra S. (2020). Functional and Dysfunctional Neuroplasticity in Learning to Cope with Stress. Brain Sci..

[129] Choi G.E., Han H.J. (2021). Glucocorticoid impairs mitochondrial quality control in neurons. Neurobiol. Dis..

[130] Gregus A., Wintink A.J., Davis A.C., Kalynchuk L.E. (2005). Effect of repeated corticosterone injections and restraint stress on anxiety and depression-like behavior in male rats. Behav. Brain Res..

[131] Johnson S.A., Fournier N.M., Kalynchuk L.E. (2006). Effect of different doses of corticosterone on depression-like behavior and HPA axis responses to a novel stressor. Behav. Brain Res..

[132] Mayer J.L., Klumpers L., Maslam S., De Kloet E.R., Joëls M., Lucassen P.J. (2006). Brief Treatment With the Glucocorticoid Receptor Antagonist Mifepristone Normalises the Corticosterone-Induced Reduction of Adult Hippocampal Neurogenesis. J. Neuroendocr..

[133] Sheline Y.I., Wang P.W., Gado M.H., Csernansky J.G., Vannier M. (1996). Hippocampal atrophy in recurrent major depression. Proc. Natl. Acad. Sci. USA.

[134] Gong Q., Yan X.-J., Lei F., Wang M.-L., He L.-L., Luo Y.-Y., Gao H.-W., Feng Y.-L., Yang S.-L., Li J. (2019). Proteomic profiling of the neurons in mice with depressive-like behavior induced by corticosterone and the regulation of berberine: Pivotal sites of oxidative phosphorylation. Mol. Brain.

[135] Suwanjang W., Wu K.L.H., Prachayasittikul S., Chetsawang B., Charngkaew K. (2019). Mitochondrial Dynamics Impairment in Dexamethasone-Treated Neuronal Cells. Neurochem. Res..

[136] Schumacher M., Weill-Engerer S., Liere P., Robert F., Franklin R., Garcia-Segura L., Lambert J., Mayo W., Melcangi C.R., Parducz A. (2003). Steroid hormones and neurosteroids in normal and pathological aging of the nervous system. Prog. Neurobiol..

[137] Gaignard P., Liere P., Thérond P., Schumacher M., Slama A., Guennoun R. (2017). Role of Sex Hormones on Brain Mitochondrial Function, with Special Reference to Aging and Neurodegenerative Diseases. Front. Aging Neurosci..

[138] Ratner M., Kumaresan V., Farb D.H. (2019). Neurosteroid Actions in Memory and Neurologic/Neuropsychiatric Disorders. Front. Endocrinol..

[139] Zorumski C.F., Paul S.M., Covey D.F., Mennerick S. (2019). Neurosteroids as novel antidepressants and anxiolytics: GABA-A receptors and beyond. Neurobiol. Stress.

[140] Grimm A., Biliouris E.E., Lang U.E., Götz J., Mensah-Nyagan A.G., Eckert A. (2015). Sex hormone-related neurosteroids differentially rescue bioenergetic deficits induced by amyloid-β or hyperphosphorylated tau protein. Cell. Mol. Life Sci..

[141] Rettberg J.R., Yao J., Brinton R.D. (2013). Estrogen: A master regulator of bioenergetic systems in the brain and body. Front. Neuroendocr..

[142] Rasgon N.L., Silverman D., Siddarth P., Miller K., Ercoli L.M., Elman S., Lavretsky H., Huang S.-C., Phelps M.E., Small G.W. (2005). Estrogen use and brain metabolic change in postmenopausal women. Neurobiol. Aging.

[143] Wroolie T.E., Kenna H.A., Williams K.E., Rasgon N.L. (2015). Cognitive Effects of Hormone Therapy Continuation or Discontinuation in a Sample of Women at Risk for Alzheimer Disease. Am. J. Geriatr. Psychiatry.

[144] Georgakis M.K., Thomopoulos T.P., Diamantaras A.-A., Kalogirou E.I., Skalkidou A., Daskalopoulou S.S., Petridou E. (2016). Association of Age at Menopause and Duration of Reproductive Period With Depression After Menopause. JAMA Psychiatry.

[145] Georgakis M.K., Kalogirou E.I., Diamantaras A.-A., Daskalopoulou S.S., Munro C.A., Lyketsos C.G., Skalkidou A., Petridou E.T. (2016). Age at menopause and duration of reproductive period in association with dementia and cognitive function: A systematic review and meta-analysis. Psychoneuroendocrinology.

[146] Gurvich C., Hoy K., Thomas N., Kulkarni J. (2018). Sex Differences and the Influence of Sex Hormones on Cognition through Adulthood and the Aging Process. Brain Sci..

[147] Zárate S., Astiz M., Magnani N., Imsen M., Merino F., Álvarez S., Reinés A., Seilicovich A. (2017). Hormone deprivation alters mitochondrial function and lipid profile in the hippocampus. J. Endocrinol..

[148] Jones T.T., Brewer G.J. (2009). Critical age-related loss of cofactors of neuron cytochrome C oxidase reversed by estrogen. Exp. Neurol..

[149] Irwin R.W., Yao J., Hamilton R.T., Cadenas E., Brinton R.D., Nilsen J. (2008). Progesterone and Estrogen Regulate Oxidative Metabolism in Brain Mitochondria. Endocrinology.

[150] Irwin R.W., Yao J., Ahmed S.S., Hamilton R.T., Cadenas E., Brinton R.D. (2010). Medroxyprogesterone Acetate Antagonizes Estrogen Up-Regulation of Brain Mitochondrial Function. Endocrinology.

[151] Zhao L., Morgan T.E., Mao Z., Lin S., Cadenas E., Finch C.E., Pike C., Mack W.J., Brinton R.D. (2012). Continuous versus Cyclic Progesterone Exposure Differentially Regulates Hippocampal Gene Expression and Functional Profiles. PLoS ONE.

[152] Uchida M., Palmateer J.M., Herson P.S., Devries A.C., Cheng J., Hurn P.D. (2009). Dose-Dependent Effects of Androgens on Outcome after Focal Cerebral Ischemia in Adult Male Mice. Br. J. Pharmacol..

[153] Barreto G., Veiga S., Azcoitia I., Garcia-Segura L.M., Garcia-Ovejero D. (2007). Testosterone decreases reactive astroglia and reactive microglia after brain injury in male rats: Role of its metabolites, oestradiol and dihydrotestosterone. Eur. J. Neurosci..

[154] Son S.-W., Lee J.-S., Kim H.-G., Kim D.-W., Ahn Y.-C., Son C.-G. (2015). Testosterone depletion increases the susceptibility of brain tissue to oxidative damage in a restraint stress mouse model. J. Neurochem..

[155] Fanaei H., Karimian S.M., Sadeghipour H.R., Hassanzade G., Kasaeian A., Attari F., Khayat S., Ramezani V., Javadimehr M. (2014). Testosterone enhances functional recovery after stroke through promotion of antioxidant defenses, BDNF levels and neurogenesis in male rats. Brain Res..

[156] Hioki T., Suzuki S., Morimoto M., Masaki T., Tozawa R., Morita S., Horiguchi T. (2013). Brain Testosterone Deficiency Leads to Down-Regulation of Mitochondrial Gene Expression in Rat Hippocampus Accompanied by a Decline in Peroxisome Proliferator-Activated Receptor-γ Coactivator 1α Expression. J. Mol. Neurosci..

[157] Baez E., Echeverria V., Cabezas R., Ávila-Rodriguez M., Garcia-Segura L.M., Barreto G.E. (2016). Protection by Neuroglobin Expression in Brain Pathologies. Front. Neurol..

[158] Reutzel M., Grewal R., Dilberger B., Silaidos C., Joppe A., Eckert G.P. (2020). Cerebral Mitochondrial Function and Cognitive Performance during Aging: A Longitudinal Study in NMRI Mice. Oxidative Med. Cell. Longev..

[159] McCullough L.D., Zeng Z., Blizzard K.K., Debchoudhury I., Hurn P.D. (2005). Ischemic Nitric Oxide and Poly (ADP-Ribose) Polymerase-1 in Cerebral Ischemia: Male Toxicity, Female Protection. Br. J. Pharmacol..

[160] Liu F., Li Z., Li J., Siegel C., Yuan R., McCullough L.D. (2009). Sex Differences in Caspase Activation After Stroke. Stroke.

[161] Gaignard P., Fréchou M., Schumacher M., Thérond P., Mattern C., Slama A., Guennoun R. (2015). Progesterone reduces brain mitochondrial dysfunction after transient focal ischemia in male and female mice. Br. J. Pharmacol..

[162] Gaignard P., Fréchou M., Liere P., Thérond P., Schumacher M., Slama A., Guennoun R. (2018). Sex differences in brain mitochondrial metabolism: Influence of endogenous steroids and stroke. J. Neuroendocr..

[163] Pike C.J., Nguyen T.-V.V., Ramsden M., Yao M., Murphy M.P., Rosario E.R. (2008). Androgen cell signaling pathways involved in neuroprotective actions. Horm. Behav..

[164] Witzig M., Grimm A., Schmitt K., Lejri I., Frank S., Brown S.A., Eckert A. (2020). Clock-Controlled Mitochondrial Dynamics Correlates with Cyclic Pregnenolone Synthesis. Cells.

[165] Lejri I., Grimm A., Hallé F., Abarghaz M., Klein C., Maitre M., Schmitt M., Bourguignon J.-J., Mensah-Nyagan A.G., Bihel F. (2019). TSPO Ligands Boost Mitochondrial Function and Pregnenolone Synthesis. J. Alzheimer’s Dis..

[166] Duarte A., Poderoso C., Cooke M., Soria G., Maciel F.C., Gottifredi V., Podesta E.J. (2012). Mitochondrial Fusion Is Essential for Steroid Biosynthesis. PLoS ONE.

[167] Rune G., Frotscher M. (2005). Neurosteroid synthesis in the hippocampus: Role in synaptic plasticity. Neuroscience.

[168] Sayeed I., Parvez S., Wali B., Siemen D., Stein D.G. (2009). Direct inhibition of the mitochondrial permeability transition pore: A possible mechanism for better neuroprotective effects of allopregnanolone over progesterone. Brain Res..

[169] Lejri I., Grimm A., Miesch M., Geoffroy P., Eckert A., Mensah-Nyagan A.-G. (2017). Allopregnanolone and its analog BR 297 rescue neuronal cells from oxidative stress-induced death through bioenergetic improvement. Biochim. Biophys. Acta (BBA)-Mol. Basis Dis..

[170] Wang T., Yao J., Chen S., Mao Z., Brinton R.D. (2019). Allopregnanolone Reverses Bioenergetic Deficits in Female Triple Transgenic Alzheimer’s Mouse Model. Neurotherapeutics.

[171] Patel M.A., Katyare S.S. (2006). Dehydroepiandrosterone (DHEA) treatment stimulates oxidative energy metabolism in the cerebral mitochondria from developing rats. Int. J. Dev. Neurosci..

[172] Patel M.A., Katyare S.S. (2007). Effect of dehydroepiandrosterone (DHEA) treatment on oxidative energy metabolism in rat liver and brain mitochondria. A dose–response study. Clin. Biochem..

[173] Grimm A., Schmitt K., Lang U.E., Mensah-Nyagan A.G., Eckert A. (2014). Improvement of neuronal bioenergetics by neurosteroids: Implications for age-related neurodegenerative disorders. Biochim. Biophys. Acta (BBA)-Mol. Basis Dis..

[174] Chen H.-B., Xu C., Zhou M.-H., Qiao H., An S.-C. (2021). Endogenous hippocampal, not peripheral, estradiol is the key factor affecting the novel object recognition abilities of female rats. Behav. Neurosci..

[175] Garcia-Segura L., Wozniak A., Azcoitia I., Rodriguez J.-R., Hutchison R., Hutchison J. (1999). Aromatase expression by astrocytes after brain injury: Implications for local estrogen formation in brain repair. Neuroscience.

